# Light microscopy of proteins in their ultrastructural context

**DOI:** 10.1038/s41467-020-17523-8

**Published:** 2020-07-31

**Authors:** Ons M’Saad, Joerg Bewersdorf

**Affiliations:** 1 Department of Cell Biology, Yale School of Medicine, New Haven, CT USA; 2 Department of Biomedical Engineering, Yale University, New Haven, CT USA; 3 Kavli Institute for Neuroscience, Yale School of Medicine, New Haven, CT USA; 4 Nanobiology Institute, Yale University, West Haven, CT USA

**Keywords:** Fluorescence imaging, Super-resolution microscopy

## Abstract

Resolving the distribution of specific proteins at the nanoscale in the ultrastructural context of the cell is a major challenge in fluorescence microscopy. We report the discovery of a new principle for an optical contrast equivalent to electron microscopy (EM) which reveals the ultrastructural context of the cells with a conventional confocal microscope. By decrowding the intracellular space through 13 to 21-fold physical expansion while simultaneously retaining the proteins, bulk (pan) labeling of the proteome resolves local protein densities and reveals the cellular nanoarchitecture by standard light microscopy.

## Abstract (web summary)

Imaging specific proteins in the ultrastructural context largely relies on correlative light/electron microscopy, but fluorophore incompatibility and registration issues limit its use. Here the authors develop an expansion microscopy method with pan-labeling of the proteome to obtain EM-equivalent light microscopy images.

## Introduction

Fluorescence microscopy has transformed the field of cell biology through its exceptional contrast and high specificity of labeling. With the advent of super-resolution microscopy, the three-dimensional (3D) distribution of specific proteins of interest can be imaged at spatial resolutions down to ~10 nm, revealing their astounding sub-cellular organization at the nanoscale^1^. Fluorescence microscopy, unlike electron microscopy, is however fundamentally prohibited from resolving the ultrastructural context of the cell: the smallest fluorescent labels, organic dyes, are ~1 nm in diameter, a size comparable to the distance between proteins in the densely crowded cellular interior^2^. Many binding sites are therefore masked or unreachable and neighboring fluorescent labels sterically hinder or self-quench each other via electron transfer or dipole-dipole interactions^3^. This limits the achievable fluorophore density and thereby the contrast and sampling necessary to resolve the crowded and complex ultrastructural context of the cell^4^. Showing specific proteins in their ultrastructural context, has therefore largely relied on correlative light/electron microscopy (CLEM). These techniques combine the resolving power and global contrast of EM with the information provided by high molecular specificity of fluorescence microscopy. While CLEM can yield information-rich images of the 3D landscape of the cell^5^, it requires highly specialized instruments and days to weeks of continuous data acquisition for a single 3D data set of a mammalian cell, thereby limiting its applicability.

Here, we demonstrate a straightforward method of imaging whole-cell ultrastructural context based on Expansion Microscopy (ExM). In ExM^6–12^, a biological sample is embedded and hybridized to a swellable poly(acrylamide/sodium acrylate) co-polymer network. By absorbing water, the gel physically expands by a factor of ~4 in all three dimensions. In iterative expansion microscopy (iExM), iteratively anchoring DNA oligo-conjugated antibodies to hydrogel networks twice yields expansion factors of typically up to 20^10^. The commonly used proteases, which digest proteins to allow for homogeneous expansion, however, result in the degradation of most cellular content. Variants of ExM such as Magnified Analysis of the Proteome (MAP) and Ultrastructure-ExM (U-ExM) have addressed this problem by preventing inter- and intra-protein crosslinks during the tissue-hydrogel hybridization step and by using anionic surfactants and heat to isotropically separate neighboring proteins^9,12^. However, despite their promise, these techniques have been limited to an expansion factor of ~4 as they are not compatible with iterative expansion.

We hypothesized that by embedding a sample in a dense hydrogel prepared with a cleavable crosslinker in a second dense superabsorbent hydrogel, entanglements between polymer chains of the first and final hydrogels will physically interlock protein-polymer hybrids in this latter polymer network, thereby preserving the proteome while iteratively expanding it. This type of polymer entanglement, first described by Sam Edwards in 1967^13^, would retain most of the cellular proteome in the final hydrogel, while simultaneously expanding the sample by about a factor of 4 × 4 = 16. The hydrogel chemistry employed in this method is reminiscent of semi-interpenetrating polymer networks (semi-IPNs), where one polymer is crosslinked and the second is linear^14^. Because of their enhanced and tunable mechanical properties, semi-IPN hydrogels have been designed to entrap cells, proteins, and small molecule drugs for controlled drug release^15–17^. In this paper, we show that the application of semi-IPNs can be extended to the entrapment of polymer-protein hybrids to increase the sample expansion factor while retaining its protein content. This strategy combined with global fluorescent labeling, which targets all separated proteins, would reveal the overall landscape of the cell with a light microscope - resembling the contrast of heavy-metal EM stains.

Based on this concept, we developed an ExM method which we named, in reference to the philosophy of labeling the whole (Greek: pan) proteome, pan-ExM. In brief (Supplementary Figs. 1 and 2), previously fixed cells cultured on coverslips are incubated in a solution of acrylamide (AAm) and formaldehyde (FA) to simultaneously prevent inter-protein crosslinks and tether proteins to the hydrogel. Next, the cells are embedded in a poly (acrylamide/sodium acrylate) co-polymer cross-linked with *N,N*′-(1,2-dihydroxyethylene)bisacrylamide (DHEBA), an acrylamide crosslinker with a cleavable amidomethylol bond. After polymerization, the sample is delipidated and denatured with sodium dodecyl sulfate (SDS) and heat. The hydrogel is then expanded ~4-fold in deionized water and embedded in a neutral polyacrylamide hydrogel cross-linked with DHEBA to maintain the gel in its expanded state during subsequent treatments. Afterwards, the gel is incubated in a solution of FA and AAm for additional anchoring of previously masked primary amines to the next hydrogel. This composite hydrogel is embedded in a third hydrogel, a poly(acrylamide/sodium acrylate) co-polymer cross-linked with *N,N*′-methylenebis(acrylamide) (BIS), a non-hydrolyzable acrylamide crosslinker. Next, sodium hydroxide is used to cleave the crosslinks of the first and second hydrogels and the sample is washed with PBS. Lastly, the hydrogel is labeled with fluorescent dyes and expanded to its final size in deionized water with an expected linear expansion factor of ~16 (Supplementary Fig. 3).

## Results

### pan-ExM reveals cellular ultrastructure

**Fig. 1:**
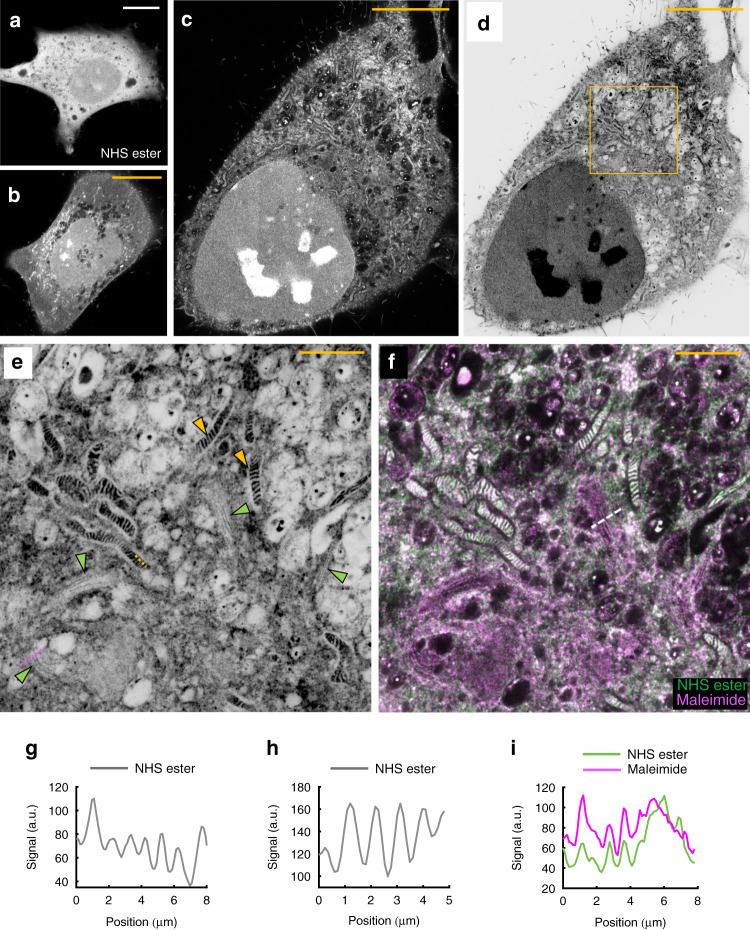
pan-ExM reveals cellular ultrastructure. **a** Non-expanded HeLa cell pan-stained with NHS ester dye. **b** HeLa cell expanded 4-fold and pan-stained with NHS ester dye. **c** pan-ExM expanded HeLa cell pan-stained with NHS ester dye. **d** Same image as **c** but shown with an inverted color table. **e** The area in the yellow box in **d** reveals hallmark cellular ultrastructure features such as mitochondrial cristae (yellow arrowheads) and Golgi cisternae (green arrowheads). **f** Same image as in **e** but showing overlay of NHS ester pan-stain channel (green) and maleimide pan-stain channel (magenta; see Supplementary Fig. 23). Representative images from 3 (**a**, **b**) and 11 (**c**–**f**) independent experiments are shown. **g** Line profile along the magenta dashed line in **e** revealing Golgi cisternae. **h** Line profile along the yellow dashed line in **e** revealing mitochondrial cristae. **i** Line profile along the dashed line in **f** revealing the change in NHS ester to maleimide staining across a Golgi stack. Panels **a**–**c** are displayed with a black-to-white color table. Panels **d**, **e** are displayed with a white-to-black color table. Yellow scale bars are not corrected for the expansion factor. Scale bars, (**a**) 10 μm, (**b**) 40 μm, (**c**, **d**) 100 μm, (**e**, **f**) 20 μm.

To test this concept, we labeled proteins in bulk with what we refer to as a pan-staining. For this first experiment we chose *N*-Hydroxysuccinimide (NHS) ester-dye conjugates, taking advantage of the abundance of primary amines on proteins (Supplementary Fig. 4a). A visual comparison of HeLa cells non-expanded, expanded once, or twice, imaged with a standard confocal microscope (Fig. 1a–d; Supplementary Fig. 5), confirms the validity of this concept: non-expanded cells show essentially a uniform staining, revealing little information. Single-expanded cells start to show the outlines of organelles such as mitochondria, but fail to show ultrastructural details. Expanding cells twice, in contrast, allows to spatially resolve details too small to be distinguished by conventional light microscopy methods in standard samples. Analogous to EM, now resolvable hallmark features such as mitochondrial cristae (Fig. 1e, yellow arrowheads) or the stacking of Golgi cisternae (Fig. 1e, green arrowheads) allow for the identification of organelles by their morphological characteristics.

### pan-ExM is compatible with conventional fluorescent labels

**Fig. 2:**
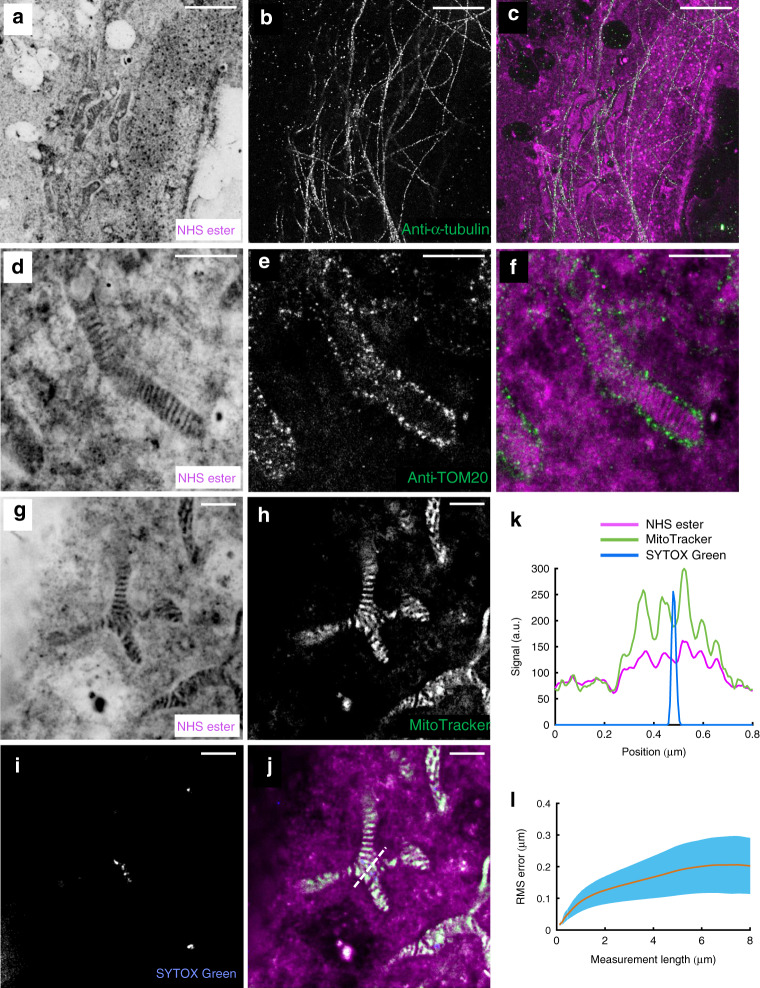
pan-ExM is compatible with standard fluorescent labeling techniques. **a** NHS ester pan-stained HeLa cell. **b** Anti-α-tubulin immunostaining in the same area. **c** Overlay of **a** and **b**. **d** NHS ester pan-stained mitochondrion. **e** Same area as in **d**, showing anti-TOM20 immunostaining and revealing the outer membrane of the mitochondrion. **f** Overlay of **d** and **e**. **g** NHS ester pan-stained mitochondrion. **h** MitoTracker Orange stain in the same area. **i** SYTOX Green stain showing DNA in mitochondrial nucleoids. **j** Overlay of **g**–**i**. **k** Line profile along the dashed line shown in **j**. Representative images from 3 (**a**–**f**) and 5 (**g**–**j**) independent experiments are shown. **l** Plot of root mean square (RMS) error over distance comparing pre- and post-pan-ExM images of microtubules (*n* = 5 cells). The orange line corresponds to the mean and blue error bars correspond to the standard deviation. Panels **a**, **d**, **g** are displayed with a white-to-black color table. Panels **b**, **e**, **h**, **i** are displayed with a black-to-white color table. Scale bars show expansion-corrected values. Scale bars, (**a**–**c**) 2 μm, (**d**–**f**) 1 μm, (**g**–**j**) 500 nm.

Importantly, our protocol is compatible with immunofluorescence labeling as well as other established chemical stainings, enabling correlative studies which combine specific and contextual pan-labeling approaches. Figure 2a–c and Supplementary Fig. 6 show microtubules labeled by an antibody against α-tubulin. Similarly, we can visualize the outer mitochondrial membrane protein TOM20 by antibody-labeling in the context of the cellular ultrastructure revealed by our pan-staining (Fig. 2d–f). It is worth pointing out that these antibodies were applied after the expansion procedure. This approach allows us to take advantage of the molecular decrowding for improved epitope accessibility and leads to a negligible label size relative to the expanded structure sizes. Furthermore, Fig. 2h shows a pan-ExM sample labeled with a live-cell small molecule dye, MitoTracker Orange, which covalently binds to proteins in the matrix of mitochondria. The MitoTracker Orange staining overlays well with the striped pattern observed in the NHS ester channel (Fig. 2g, j, k), confirming that the latter represents the mitochondrial matrix. DNA-intercalating dyes such as SYTOX Green are equally compatible with pan-ExM and label, next to the nucleus, mitochondrial nucleoids (Fig. 2i, j). The resolution achieved with the used standard confocal microscope is good enough to reveal the partial exclusion of proteins from the nucleoids (as represented by NHS-ester and MitoTracker staining; Fig. 2k) which have been reported to be about 110 nm in diameter^18^.

### pan-ExM reveals nuclear ultrastructure

**Fig. 3:**
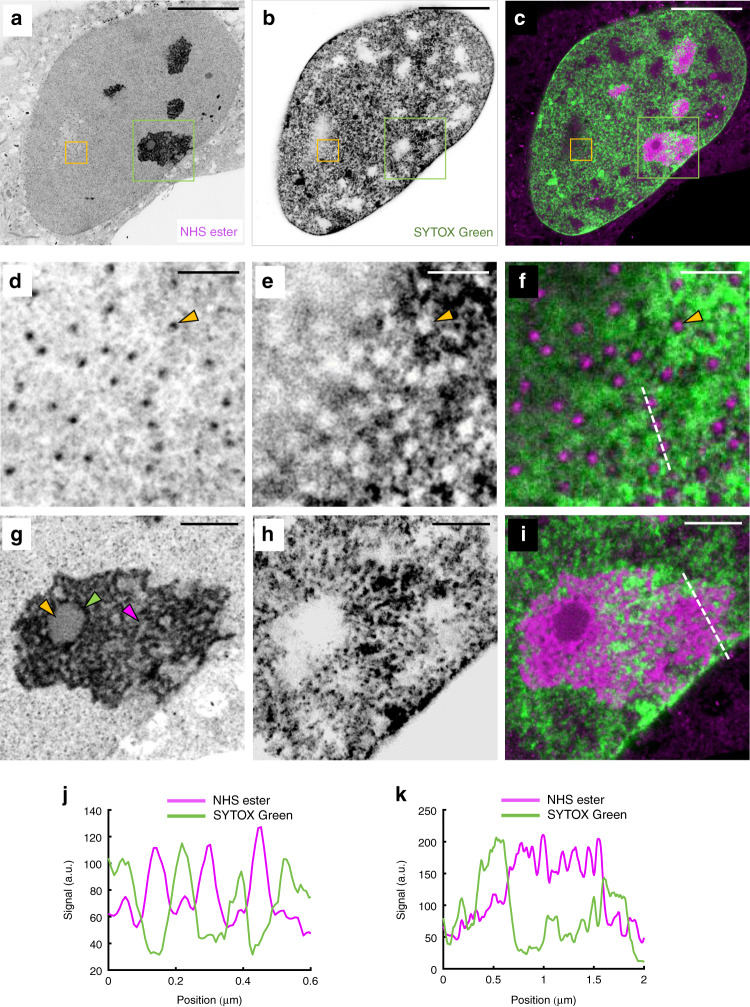
pan-ExM reveals nuclear architecture in interphase. **a** Image of NHS ester pan-stained U-2OS cell in interphase. **b** SYTOX Green nucleic acid stain image of the same area. **c** Overlay of **a** and **b**. **d**–**f** Magnified view of the areas outlined by the yellow boxes in **a**–**c** showing amine-rich regions corresponding to nuclear pore complexes (NPCs) which coincide with circular channels excluding chromatin in the SYTOX Green image. The yellow arrowheads point at one NPC and the corresponding chromatin channel. **g**–**i** Magnified view of the areas outlined by the green boxes in **a**–**c** showing ultrastructural details of a nucleolus. The yellow, green and magenta arrowheads point at the fibrillar center (FC), the dense fibrillar component (DFC) and the granular component (GC), respectively. Representative images from 5 (**a**–**i**) independent experiments are shown. **j** Line profile along the dashed line shown in **f**. **k** Line profile along the dashed line shown in **i**. Panels **a**, **b**, **d**, **e**, **g**, **h** are displayed with a white-to-black color table. All scale bars are corrected for the determined expansion factor. Scale bars, (**a**–**c**) 5 μm, (**d**–**f**) 250 nm, (**g**–**i**), 1 μm.

Focusing on the cell nucleus, we find that SYTOX Green produces a bright nuclear staining (Fig. 3a–c). Nuclear pore complexes appear as bright spots in the NHS ester pan-staining (also visible in Fig. 2a) and are clearly correlated with nuclear regions showing reduced SYTOX Green staining (Fig. 3d–f, j; Supplementary Fig. 7). This observation has been made by EM^19^ and super-resolution microscopy^20^ before. Similarly, the SYTOX Green staining is partially excluded from nucleoli identifiable by their strong NHS ester pan-staining (Fig. 3g–i, k; Supplementary Fig. 7). Nucleoli subcompartments such as the granular component, fibrillar center, and dense fibrillar components can easily be resolved^21^. Intriguingly, we can observe areas of strong nuclear acid staining in close vicinity of, but not overlapping with, the fibrillar components of the nucleoli (Fig. 3g–i, k; Supplementary Figs. 7 and 8 show nucleoli in U-2OS and HeLa cells, respectively).

### pan-ExM reveals mitotic cell ultrastructure

**Fig. 4:**
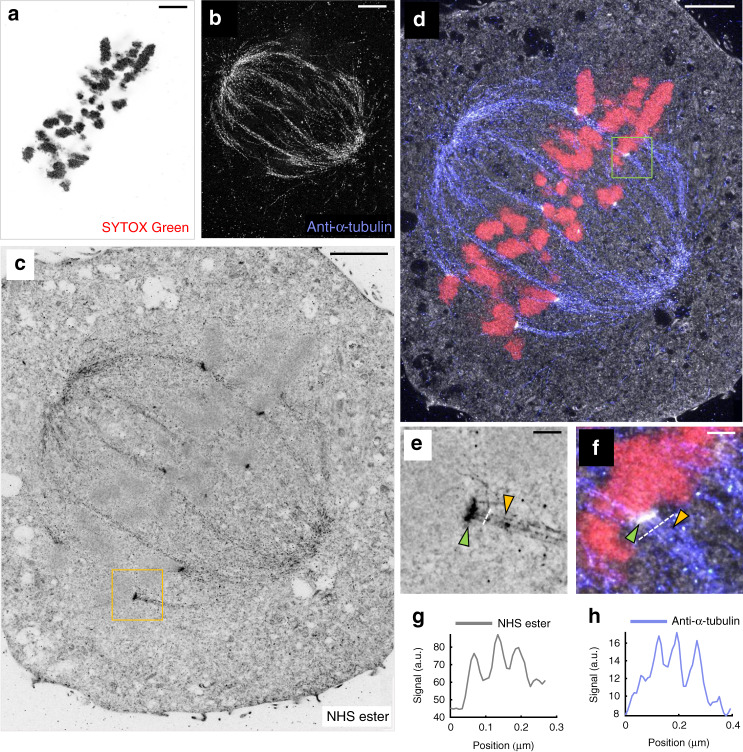
pan-ExM reveals mitotic cell ultrastructure. **a** SYTOX Green channel of a mitotic U-2OS cell revealing chromosomes. **b** Anti-α-tubulin immunostaining in the same area. **c** NHS ester pan-staining in the same area. **d** Overlay of **a**–**c**. **e** Magnified image of the area outlined by the yellow box in **c**. **f** Magnified image of the area outlined by the green box in **d**. The yellow arrowheads highlight individual microtubules within microtubule bundles, the green arrowheads point at kinetochores. Representative images from 3 (**a**–**f**) independent experiments. **g** Line profile along the dashed line shown in **e**. **h** Line profile along the dashed line shown in **f**. Panels **a**, **c**, **e** are displayed with a white-to-black color table. Panel **b** is displayed with a black-to-white color table. All scale bars are corrected for the determined expansion factor. Scale bars, (**a**–**d**) 2 μm, (**e**, **f**) 300 nm.

Labeling a mitotic U-2OS cell with α-tubulin antibody and SYTOX Green in addition to our NHS ester pan-staining, reveals the mitotic spindle with individual filaments resolved within microtubule bundles (Fig. 4). Interestingly, NHS ester pan-staining appears to boost signal from α-tubulin antibody labeling, when applied after. This suggests that NHS ester also labels primary amines on antibodies, offering additional signal amplification. Additionally, kinetochores, easily identifiable by their association to chromosomes and microtubule ends, become clearly visible by their bright NHS ester signal (Fig. 4e, f). Individual chromosomes are resolvable in the SYTOX Green channel revealing fine chromatin fibers extending from the chromosomes (Fig. 4a) which are consistent with the polymer brush-like architecture model of mitotic chromosomes^22^. Similar chromatin organization can be observed in the mitotic HeLa cell image shown in Supplementary Fig. 9. Another hallmark structure of cell division, the cleavage furrow, also shows strong contrast in NHS ester pan-stained samples, revealing the clearly discernible midbody and the very dense matrix of the Flemming body (Supplementary Fig. 10).

### Expansion factor and homogeneity

To determine the achieved linear expansion factors, we imaged SYTOX Green-stained HeLa cell nuclei in non-expanded samples and samples expanded using our standard protocol (see Methods) and compared the average nuclear cross-sectional area in both cases. On average, we obtained an expansion factor of 14.3 ± 0.9 (mean ± s.d.; *N* = 6 experiments; *n* = 21–58 nuclei per experiment; see Methods and Supplementary Fig. 11b, c). We next investigated the homogeneity of the expansion by imaging three different cellular structures, mitochondria, microtubules, and the nucleus, before and after expansion. Briefly, we registered the corresponding data sets with a similarity transform and then registered the similarity-corrected post-expansion image to the pre-expansion image with a B-spline non-rigid transform to detect local heterogeneities in expansion^6^ (see Methods, Supplementary Fig. 12). Global expansion factors determined by this approach were 15.6 ± 0.3 (nuclei), 15.8 ± 0.7 (microtubules) and 17.1 ± 0.7 (mitochondria) (*n* = 5 fields of view, *N* = 1 sample, each; Supplementary Fig. 11a) and were largely in agreement with the value listed above. For structures 8–20 μm apart, we measured root-mean-square (RMS) errors of ~200–670 nm, corresponding to ~2.5–3.2% of the measurement length (Fig. 2l, Supplementary Fig. 12). The determined RMS error distributions are comparable to results published for other protease-free ExM methods^9,12^. To investigate if anisotropic stretching during sample handling contributes to these results, we replaced the similarity transform with an affine transform (Supplementary Fig. 12). The relative RMS errors dropped as a result to ~1.5–1.7% suggesting that mechanical deformations from handling the sample are responsible for about half of the observed RMS error in the similarity transform data.

### Validation of pan-ExM at the nanoscale

**Fig. 5:**
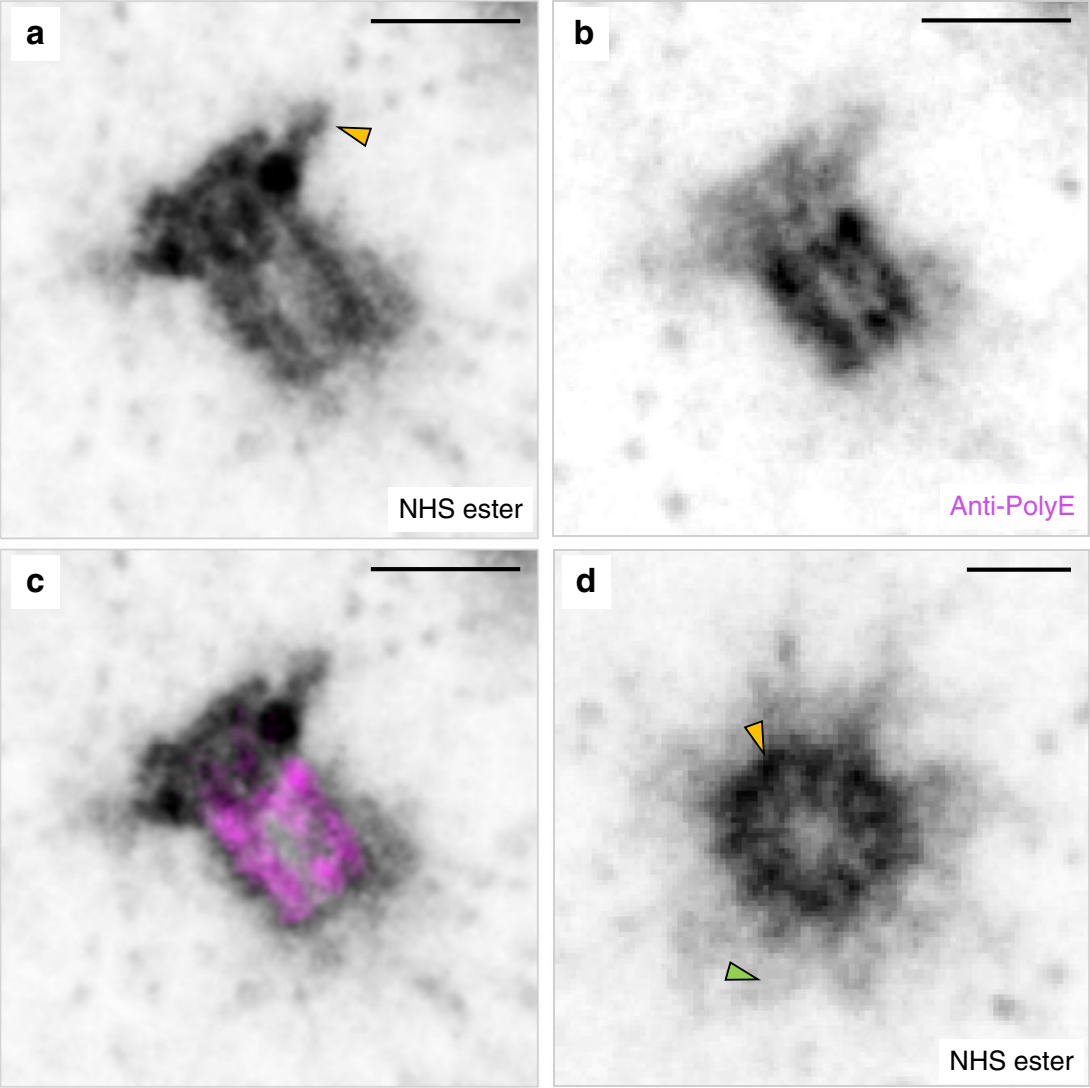
pan-ExM reveals ultrastructural details of centrosomes. **a** Lateral view of a NHS-ester pan-labeled mature centriole in a pan-ExM processed U-2OS cell revealing subdistal appendages (yellow arrowhead). **b** Anti-polyglutamate chain (polyE) immunostaining in the same area revealing three distinct polyglutamylated microtubule triplets. **c** Overlay of **a** and **b**. **d** Axial view of a different NHS-ester pan-stained mature centriole revealing microtubule triplets (yellow arrowhead) and pericentriolar material (PCM) (green arrowhead). Representative images from 4 (**a**, **d**) and 1 (**b**, **c**) independent experiments. Panels **a**, **b**, **d** are displayed with white-to-black color tables. All scale bars are corrected for the determined expansion factor. Scale bars, (**a**–**c**) 200 nm, (**d**) 100 nm.

pan-ExM clearly can resolve ultrastructural features which are not accessible in conventional fluorescence microscopy. Figure 5, for example, shows two centrioles in human U-2OS cells, one in a lateral orientation (Fig. 5a–c) and one in an axial orientation (Fig. 5d). We observed that centrioles, which are massive protein complexes, are labeled very brightly by NHS ester pan-staining (Supplementary Fig. 13). Not only does pan-ExM reveal the dense hollow barrel geometry of the centriole but also its subdistal appendages, distal appendages, the nine-fold symmetry of microtubule triplets (Supplementary Fig. 14), and the pericentriolar material (PCM) surrounding the centrosome previously identified in EM data^23^. 3D image stacks of the centrosome (Supplementary Movies 1 and 2) also reveal the centriolar cartwheel structure and the procentrioles that associate with mature centrioles. When we immuno-labeled polyglutamylated tubulin, we observed that it labeled the centriolar wall on the central core (Fig. 5c) in agreement with previous reports^12,24^. Quantifying centriole roundness yielded a value of 0.91 ± 0.05 (mean ± s.d.; *n* = 8; Supplementary Fig. 15a), supporting the nanoscale isotropy of expansion.

**Fig. 6:**
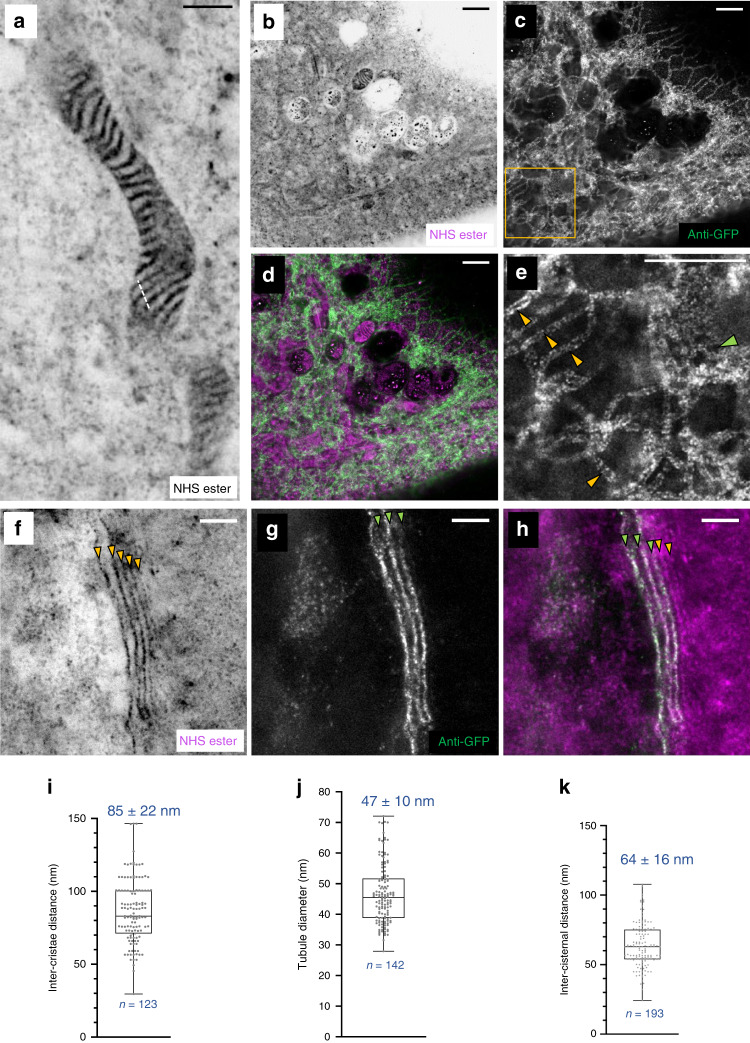
pan-ExM reveals ultrastructural details of cellular organelles. **a** NHS ester pan-stained mitochondrion in a NHS ester pan-stained HeLa cell. **b** NHS ester pan-stained HeLa cell expressing ER-membrane localized Sec61β-GFP. **c** Anti-GFP label in the same area revealing the ER. **d** Overlay of **a** and **b**. **e** Magnified image of the area outlined by the yellow box in **b**, revealing individual ER tubules clearly resolved as hollow tubules (yellow arrowheads) and a dense network of ER tubules (green arrowhead). **f** STED super-resolution image showing an NHS ester pan-stained Golgi stack in a ManII-GFP expressing HeLa cell. The yellow arrowheads show five distinct Golgi cisternae. **g** Anti-GFP STED image of the same area. The green arrowheads show three distinct Golgi cisternae. **h** Overlay of **f** and **g**. The green arrowheads point at ManII GFP-positive Golgi cisternae, the yellow ones at two ManII GFP-negative Golgi cisternae. Representative images from 11 (**a**), 2 (**b**–**e**), and 3 (**f**–**h**) independent experiments. **i** Distribution of distances between neighboring mitochondrial cristae (*n* = 123 line profiles, *N* = 4 independent experiments) calculated from cross-sections like those along the dashed line shown in **a**. **j** Distribution of ER tubule diameters (*n* = 142 cross-sections, *N* = 2 cells from 1 independent experiment). **k** Inter-cisternal distance distribution in Golgi stacks (*n* = 193 line profiles, *N* = 3 independent experiments). Medians and interquartile ranges are shown with whiskers drawn down to the minimum and maximum values. Means ± standard deviations are reported. Panels **a**, **b**, **f** are displayed with a white-to-black color table. Panels **c**, **e**, **g** are displayed with a black-to-white color table. All scale bars are corrected for the determined expansion factor. Scale bars, (**a**) 500 nm, (**b**–**e**) 1 μm, (**f**–**h**) 250 nm.

To further investigate structural preservation of features at the sub-organelle scale, we quantified the distance between mitochondrial cristae (Fig. 6a, i). The determined expansion-corrected distance of 85 ± 22 nm (mean ± s.d.) is in good agreement with previously published observations in living HeLa cells^25^. We next investigated the preservation of the endoplasmic reticulum (ER) in HeLa cells. While the ER did not show a characteristic NHS-ester pan-staining that would make it directly identifiable, we could visualize it by overexpressing ER-membrane localized Sec61β-GFP and immunolabeling with an anti-GFP antibody (Fig. 6b–e), a strategy which we have previously employed successfully in optical super-resolution studies^1,26^. pan-ExM clearly resolves the two sides of ER tubules using a standard confocal microscope (Fig. 6e) as well as the inner and outer membrane of the nuclear envelope (Supplementary Fig. 16). The superior optical resolution of a STED super-resolution microscope applied to these samples reveals distinct clustering of the antibody staining (Supplementary Fig. 17). The expansion-corrected diameter of these tubules (47 ± 10 nm, mean ± s.d.; Fig. 6j) was slightly smaller than diameters determined previously by super-resolution light microscopy of non-expanded COS-7 cells^26^ but consistent with diameters of ER tubules determined by EM^27^. Furthermore, we overexpressed the Golgi protein mannosidase II (ManII) fused to GFP in HeLa cells, labeled it with an anti-GFP antibody after applying our pan-ExM protocol and imaged it with a STED super-resolution microscope (Fig. 6f–h). It is known that ManII is located primarily in the medial cisternae of the Golgi and, when overexpressed, also in cis cisternae^1^. Consistent with this observation, we find the ManII stain to highlight 3 cisternae located at one side of the Golgi stack (Fig. 6f–h). The immunolabeled Golgi cisternae could be easily discerned by eye and overlaid well with similar structures visible in the NHS ester pan-channel. Quantifying the distance between neighboring Golgi cisternae (using conventional confocal data sets) yielded a value of 64 ± 16 nm (mean ± s.d.; *n* = 193; Fig. 6k), consistent with EM data^28^.

### Polymer entanglement as a mechanism for pan-ExM expansion

We further tested if proteins indeed are retained throughout our sample preparation procedure. For this purpose, we compared fluorescence signal levels of antibodies applied without expansion and after one or two expansion steps. The quantification shows no decrease in signal (Supplementary Fig. 18), suggesting the good protein retention capabilities of our protocol. To further investigate the protein retention mechanism, we performed an additional experiment comparing structural preservation of centrioles using our standard protocol and one where we reduced possible Schiff base groups on proteins with sodium borohydride after denaturation^29^. Schiff bases are formed as a result of formaldehyde reacting with nucleophiles on proteins and they can react covalently with acrylamide monomers^30,31^. The denaturation step is located between the polymerization of the first gel and the embedding in the second gel. Quenching reactive Schiff bases should therefore prevent crosslinking between the two gels. Additionally, we refrained from using the post-fixation step which is applied after the second gel embedding step of the standard protocol, further reducing possible crosslinking. Supplementary Fig. 15 shows the quantification of centriole roundness and length/diameter ratios. Centriole roundness between the two conditions did not differ significantly (*p* = 0.3) and the observed small difference of 15% in the length/diameter ratio distributions was barely statistically relevant (*p* = 0.047). The lack of substantial quantitative differences between the two protocols is further supported by the visual similarity between overview pan-ExM images of HeLa cells expanded following the two different protocols (compare Supplementary Fig. 19 with Fig. 1c). We conclude that while we cannot exclude a minor role of crosslinking between the gels, it does not play a major role and entanglement therefore seems to be the dominating mechanism responsible for the observed protein retention in pan-ExM.

### pan-ExM is compatible with 3D imaging

**Fig. 7:**
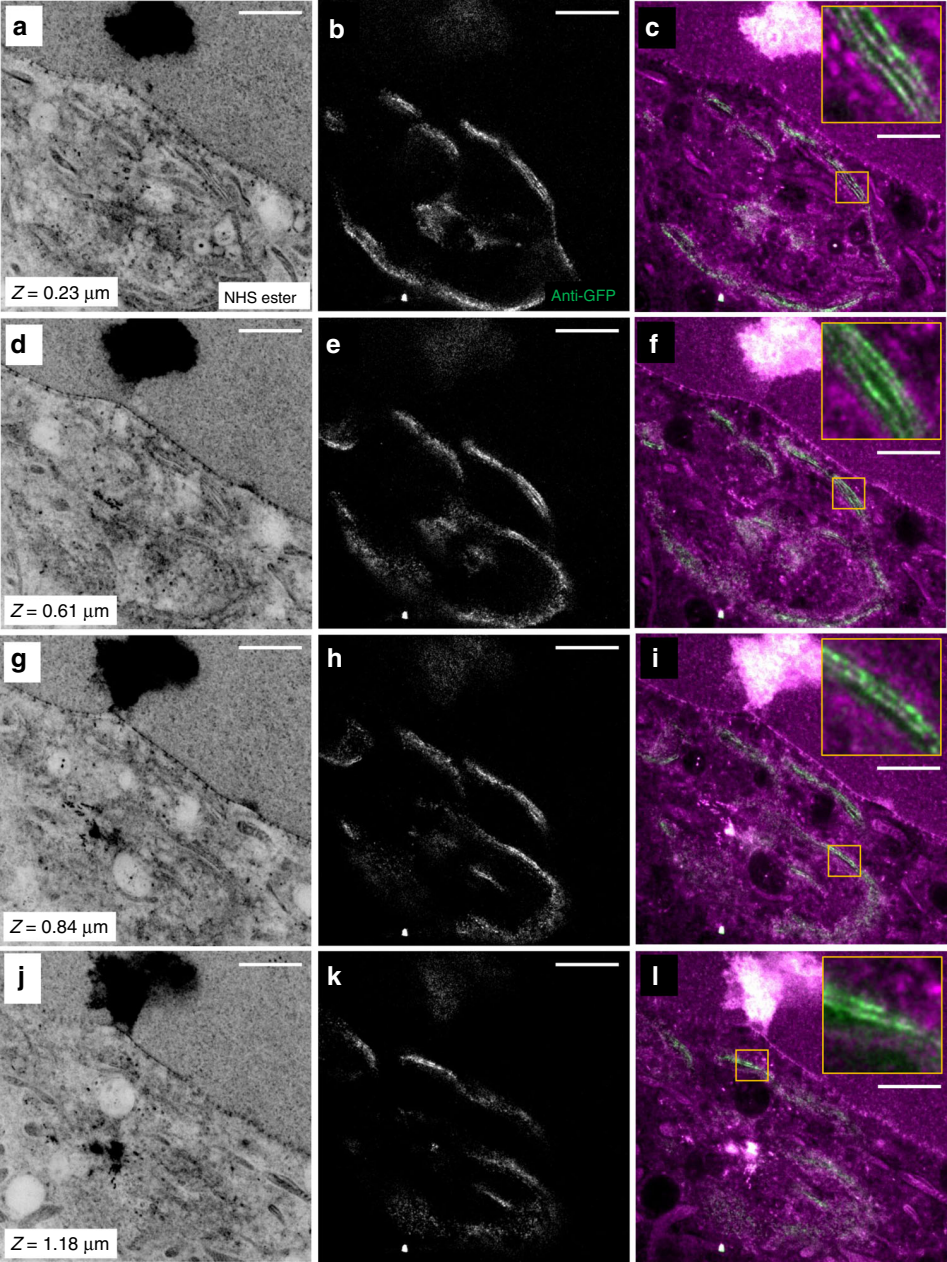
pan-ExM is compatible with 3D imaging. Images from a 3D image stack featuring the Golgi complex next to the nucleus in a HeLa cell expressing Golgi-localized ManII-GFP. **a**, **d**, **g**, **j** NHS ester images at axial positions 0.23, 0.61, 0.84 and 1.18 μm, respectively, displayed with a white-to-black color table. **b**, **e**, **h**, **k** Anti-GFP images of the same fields of view as **a**, **d**, **g**, **j** displayed with a black-to-white color table. **c**, **f**, **i**, **l** Overlay of the NHS ester and anti-GFP images. Representative images from 5 (**a**–**l**) independent experiments. The insets show the zoomed-in yellow boxes and reveal individual ManII-positive Golgi cisternae. Panels **b**, **e**, **h**, **k** were corrected for crosstalk (see Methods). Scale bar and axial positions are corrected for the determined expansion factor. Scale bars (**a**–**l**) 2 μm.

Importantly, pan-ExM is fully compatible with 3D imaging, as demonstrated by the confocal 3D image stacks shown in Fig. 7, Supplementary Fig. 20 and Supplementary Movies 3–5. The intricate structure of the convoluted Golgi ribbon and stacking of multiple cisternae is clearly revealed at all axial positions even with a conventional confocal microscope (Fig. 7; Supplementary Fig. 21). This organization has to our knowledge never before been resolved by conventional light microscopy.

### Differential pan-staining of proteins

**Fig. 8:**
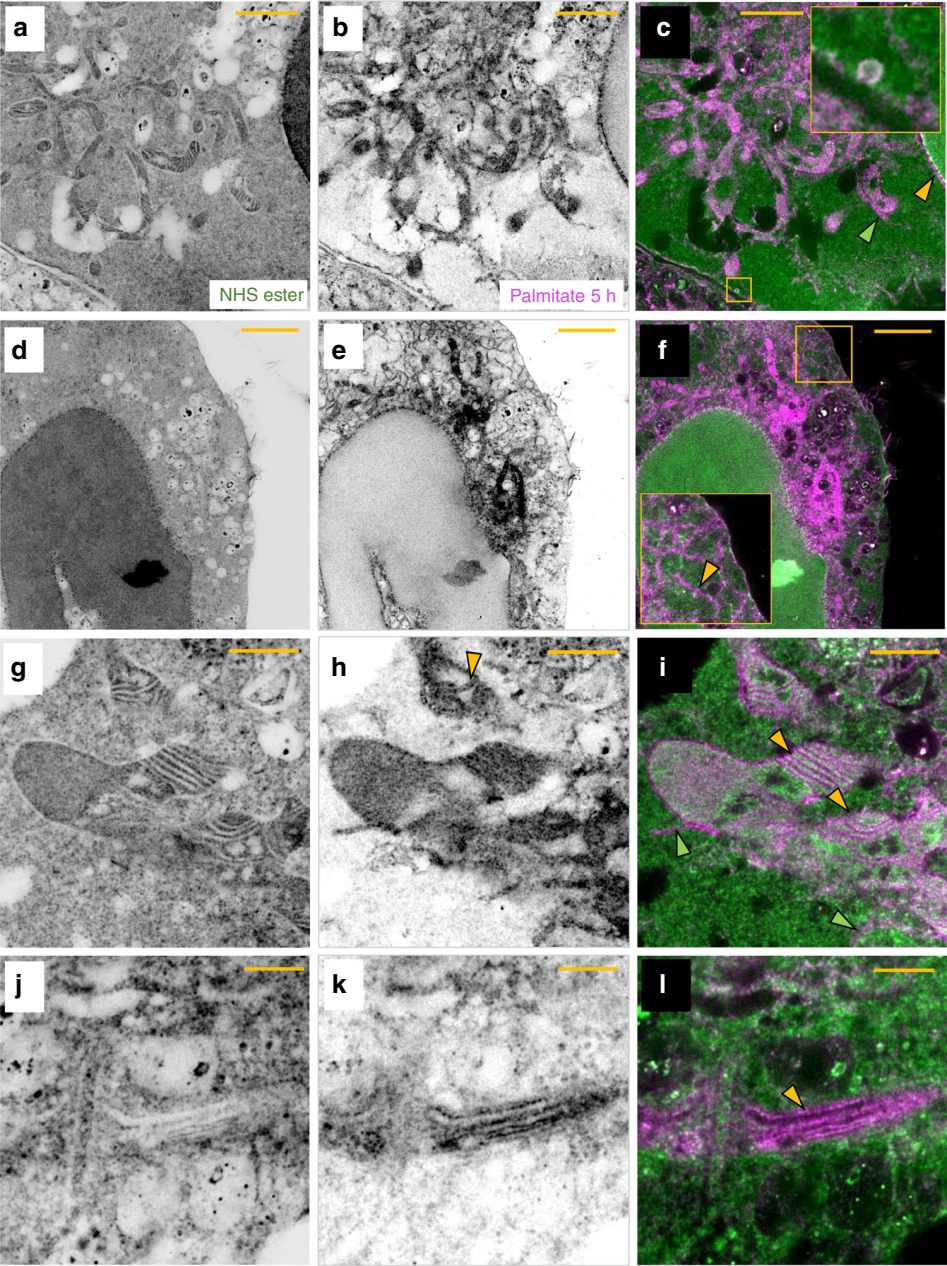
Differential pan-staining reveals compartmentalized palmitate distribution. **a** NHS ester pan-stained HeLa cell. **b** Palmitate pan-staining corresponding to the same area as shown in **a**. **c** Overlay of **a** and **b**. The area in the yellow box is shown in the inset and reveals a vesicular structure resembling a clathrin-coated pit. The yellow arrowhead points at the palmitate-rich nuclear envelope. The green arrowhead points at a tubular structure resembling an ER tubule near a mitochondrion. **d** NHS ester pan-stained HeLa cell. **e** Palmitate pan-staining corresponding to the same area as shown in **d**. **f** Overlay of **d** and **e**. The area in the yellow box is shown in the inset and reveals ER tubules (yellow arrowhead). **g** NHS ester pan-stained image of a HeLa cell showing mitochondria. **h** Palmitate pan-staining corresponding to the same area as shown in **g**. The yellow arrowhead points at a mitochondrial crista with two palmitate-rich membranes. **i** Overlay of **g** and **h**. The yellow arrowheads point at mitochondrial cristae and the green arrowheads point at tubule-like structures resembling ER tubules. **j** NHS ester pan-stained image of a HeLa cell showing a Golgi stack. **k** Palmitate pan-staining corresponding to the same area as shown in **j**. **l** Overlay of **j** and **k**. The yellow arrowhead points at a palmitate-rich Golgi cisterna. Representative images from 2 (**a**–**l**) independent experiments. Panels **a**, **b**, **d**, **e**, **g**, **h**, **j**, **k** are displayed with a white-to-black color table. Yellow scale bars are not corrected for the expansion factor. Scale bars, (**a**–**c**) 30 μm, (**d**–**f**) 50 μm, (**g**–**i**) 20 μm, (**j**–**l**) 10 μm.

One of the unique powers of pan-ExM is its capability to reveal the compartmentalization of biomolecule classes identifiable by a particular pan-staining such as NHS ester. Pan-stainings are, however, not restricted to NHS ester and other pan-stainings should show different distributions. For example, using metabolic incorporation of palmitic acid azide, one can use alkyne dyes to label palmitoylated proteins post-expansion (Supplementary Fig. 4c). Given that these proteins are insoluble in water, we expect an enriched staining in the vicinity of lipid membranes in the cell. Performing this experiment indeed produced a strong signal at the location of the plasma membrane and membrane-bound organelles (Fig. 8; Supplementary Fig. 22). Both labels are strongly enriched on structures resembling clathrin-coated pits (inset in Fig. 8c). The difference in the mitochondrial staining patterns between palmitate and NHS ester pan-stainings is particularly striking (Fig. 8i; Supplementary Fig. 22f). We note that this pattern is not visible in non-expanded cells pan-stained with the same labels (Supplementary Fig. 22c), emphasizing the role of molecular de-crowding in revealing the spatial compartmentalization of protein classes.

As another example of a pan-staining, we tested dye-conjugated maleimide (Supplementary Fig. 4b) which can reveal cellular domains with high cysteine-rich protein content. Supplementary Figs. 23 and 24 show that the maleimide staining highlights in particular the Golgi complex, most likely due to the high levels of palmitoyltransferases in the Golgi which have cysteine-rich domains^32^. The combination with NHS ester labeling (Fig. 1f and Supplementary Fig. 23) shows intriguing patterns of differential staining which provide information well beyond the monochromatic, EM-like contrast achievable with a single pan-staining - analogous to Haemotoxylin and Eosin (H&E) staining but on the nanoscale.

## Discussion

The data presented here demonstrates, from our perspective, pan-ExM’s potential to revolutionize light microscopic imaging of cellular structures. pan-ExM combines, (i), 13 to 21-fold linear expansion (obtained through iterative expansion), and (ii), protein retention through direct anchoring of proteins to the first hydrogel and transfer of entrapped proteins to the final expansion hydrogel by polymer chain entanglement. While these individual techniques have demonstrated merit, we see their true power in their combination with labeling the whole proteome: staining the cell with NHS ester dyes shifts from a largely pointless exercise in a non-expanded cell (Fig. 1a) to a technique with EM-like contrast, capable of revealing nanoscopic structural hallmarks that allow users to identify organelles without the need for specific staining (Fig. 1c, Supplementary Figs. 22, 23, 24). While the obtained resolution does not reach the level of EM, its straightforward combination with single, or multiple, specific labels stands in stark contrast to the complex protocols of immuno-gold EM or CLEM. Our sample preparation is completed in a few days; subsequent imaging takes less than a minute (2D image) to tens of minutes (3D data set; Supplementary Movie 2) per cell on a conventional light microscope. We anticipate that light-sheet microscopes or other instruments optimized for ExM^33^ will accelerate future pan-ExM data acquisition substantially.

We have shown here, using anti-GFP, anti-α-tubulin, anti-PolyE and anti-TOM20 antibodies, that pan-ExM is compatible with immunolabeling. However, some antibodies we tested did not work as they were not compatible with glutaraldehyde fixation or protein denaturation. We anticipate that this caveat can be overcome in the future by (i) focusing on antibodies which have been shown to work well in western blots due to the commonality of the denaturing step and (ii) developing fixation protocols that preserve ultrastructure without compromising protein epitope integrity. We ruled out the possibility that base hydrolysis of DHEBA crosslinker, necessary for the dissolution of the first and second hydrogels, has a detrimental effect on antibody labeling efficiency. We incubated a once-expanded sample prepared with the non-hydrolyzable crosslinker N,N′-Methylenebisacrylamide (BIS) with sodium hydroxide for 1 h prior to antibody labeling and found no significant decrease in anti-α-tubulin and anti-TOM20 signal compared to a control sample. We also validated that the treatments used to cleave other commercially available crosslinkers (e.g., N,N′-Diallyltartramide (DATD) and N,N′-Bis(acryloyl)cystamine (BAC); Supplementary Fig. 25) do not decrease antibody labeling efficiency. These alternative crosslinkers could be used for additional rounds of expansion in the future.

Resolution can be best evaluated by the structures that can be resolved. The presented examples demonstrate that we consistently resolve structures that were only about 30–100 nm apart pre-expansion. The lateral resolution of the used confocal microscope is in the 250–300 nm resolution range using a high-numerical aperture objective. Correcting this value by the measured expansion factors of ~13–21, suggests an effective expansion-corrected PSF size of 12–23 nm. Labeling samples post expansion, as usually done in our protocol, has the advantage that the label size plays essentially no role: a primary and secondary antibody pair spans at maximum a distance of 25 nm. Correcting the final image by the expansion factor shrinks this distance down to less than 2 nm. Overall, we therefore believe that it is safe to assume that the achievable lateral resolution of pan-ExM in combination with a high-end confocal microscope is in the 20–30 nm range. Combined with STED microscopy, this resolution can shrink by another factor of ~5^26^. As shown in Supplementary Fig. 26, this increase in resolution allows, for example, to not only resolve individual cristae but to distinguish the two sides of a single crista with a light microscope. The practical limit on resolution is therefore currently not the optical resolution or the global expansion factor, but our limited understanding of the expansion factor on the molecular size scale. It remains to be investigated down to which size scale structures are preserved in pan-ExM. Based on our protocol, we expect that proteins lose their tertiary structure and unfold at least partially during the sample preparation process. At the tens-of-nanometer scale, our data shows that microtubules, centrioles, and membrane-bound organelles such as mitochondria, the Golgi complex and the ER are represented well after expansion. On the other extreme: lipid droplets, having a lipid core, are dissolved by the used detergents, leaving only a shadow image of their former shape. Future systematic studies, refinements to the protocols and engineered probes, such as cross-linkable lipid labels^34,35^ and reversible protein crosslinking reagents, will answer these questions and promise further improvements.

On the positive side, pan-ExM leads to substantial molecular decrowding (~16^3^ ≈ 4000 fold by volume) of the sample. This effect is key to pan-ExM’s success: the average distance of 3 nm between proteins in the native cell becomes about 50 nm after expansion and the space in between proteins becomes easily accessible by probes even as large as antibodies (6–12 nm in size). Furthermore, the expansion protocol most likely disrupts protein clusters through denaturation, revealing previously inaccessible binding sites.

It is worth pointing out that, in contrast to optical super-resolution microscopy techniques which are ultimately limited in resolution by the size of the fluorescent labels, ExM is not constrained by label-size (if labeling is applied after expansion). Moreover, problems with labeling density which are a major limitation in optical super-resolution microscopy get alleviated: steric hindrance and fluorescence quenching in densely labeled samples become essentially irrelevant after 13- to 21-fold expansion. On the contrary, the created gaps between endogenous molecules provide space for potential biochemical amplification of the label^36^, boosting the sensitivity of the technology.

The fact that many organelles can easily be identified by eye through their characteristic NHS ester stain suggests that pan-ExM is well-suited for automated segmentation and classification algorithms to identify organelles of interest. Training of machine learning algorithms can be facilitated by additional imaging channels showing specific stains of the target organelle, for example MitoTracker for mitochondria, during the learning phase.

The pan-staining approach can be easily generalized to many sub-proteomes. We have demonstrated NHS ester, maleimide, and palmitate pan-stainings in this work (Supplementary Fig. 4). Other options could include phosphorylated and glycosylated proteins. We believe that the combination of two or more of these pan-stainings has great potential for automated segmentation of organelles and other subcellular structures of interest, and can also reveal how different sub-proteomes are distributed in the cell. We emphasize that the success of our pan-staining approach fundamentally benefits from two key strengths: high levels of protein retention and molecular decrowding provide access to large numbers of target sites; high levels of expansion offer the effective spatial resolution required to identify structures and their cellular context.

## Methods

### General comments

Please see Supplementary Tables 1–3 for an overview of the reagents and materials used in this work.

### Coverslip preparation

Before plating HeLa or U-2OS cells, 12-mm round glass coverslips (Electron Microscopy Sciences, catalog no. 72230-01) were cleaned in a sonic bath (Bronson) submerged in 1 M KOH (Macron Fine Chemicals; catalog no. 6984-04) for 15 min and then rinsed with MilliQ water three times. Glass was then sterilized with 100% ethanol, incubated with 5 µg/mL fibronectin (Sigma-Aldrich, catalog no. F1141) for 30 min, and rinsed with sterile phosphate-buffered saline (PBS; Gibco, catalog no. 10010023) before adding media and cells.

### Cell culture

HeLa and U-2OS cells were grown in Dulbecco’s modified Eagle medium (DMEM; Gibco, catalog no. 21063029), supplemented with 10% fetal bovine serum (FBS; Gibco, catalog no. 10438026), and 1% mL/L penicillin-streptomycin (Gibco, catalog no. 15140122) at 37 °C with 5% CO_2_. Cells were passaged twice to three times a week and used between passage number 2 and 20. Passaging was performed using 1× PBS and 0.05% Trypsin-EDTA (Gibco, catalog no. 25300054). Approximately 24 h before fixation, cells were seeded on fibronectin-coated glass coverslips at ~65,000 cells per well.

### Plasmids

For labeling the medial Golgi in HeLa cells, GFP-ManII was expressed. GFP-ManII was made from pEGFP-N1 (Takara Bio Inc.) to include amino acids 1-137 of mouse MAN2A1 fused to GFP, such that GFP is expressed in the Golgi lumen. For labeling the ER membrane in HeLa cells, mEmerald-Sec61-C-18 was expressed. mEmerald-Sec61-C-18 was a gift from the late Michael Davidson (Florida State University, Tallahassee, FL; Addgene plasmid # 54249; referred to as GFP-Sec61β).

### Transfection

GFP-ManII and GFP-Sec61β expression in HeLa cells used DNA transfection by electroporation^1^. DNA was introduced into the cells using a NEPA GENE electroporation device. Approximately 1 million cells were rinsed in Opti-MEM (Gibco, catalog no. 31985070) and then resuspended in Opti-MEM with 10 μg DNA in an electroporation cuvette with a 2-mm gap (Bulldog Bio, catalog no. 12358346). Cells were electroporated with a poring pulse of 125 V, 3-ms pulse length, 50-ms pulse interval, 2 pulses, with decay rate of 10% and + polarity; followed by a transfer pulse of 25 V, 50-ms pulse length, 50-ms pulse interval, 5 pulses, with a decay rate of 40% and ± polarity. After electroporation, the cells were seeded on fibronectin-coated coverslips (see Coverslip preparation). Samples were fixed 24–36 h after electroporation.

### MitoTracker orange staining

Live HeLa cells were incubated with 0.5 µM of MitoTracker Orange CMTMRos (Invitrogen, catalog no. M7510) for 30 min at 37 °C and 5% CO_2_. Next, the cells were washed three times with cell media and fixed immediately after.

### Cell fixation

Cells were fixed with 3% formaldehyde (FA) and 0.1% glutaraldehyde (GA) (Electron Microscopy Sciences, catalog nos. 15710 and 16019, respectively) in 1× PBS for 15 min at RT. Samples were rinsed three times with 1× PBS and processed according to the pan-ExM protocol immediately after.

### pan-ExM reagents

Acrylamide (AAm; catalog no. A9099), *N,N′*-(1,2-dihydroxyethylene)bisacrylamide (DHEBA; catalog no. 294381), *N,N*′-Cystaminebisacrylamide (BAC; catalog no. 9809) were purchased from Sigma-Aldrich. Three different batches of sodium acrylate (SA) were used. The first batch (catalog no. 408220, lot no. MKCF0390) was purchased from Sigma-Aldrich. The second and third batches (catalog no. sc-236893C, lot nos. H3019 and L0619) were purchased from Santa Cruz Biotechnology. We noticed significant batch-to-batch variability in SA purity. To verify that SA was of acceptable purity, 38% (w/v) solutions were made in water and checked for quality^37^. Only solutions that were light yellow were used. Solutions that were yellow and/or had a precipitate were discarded. *N,N′*-methylenebis(acrylamide) (BIS; catalog no. J66710) was purchased from Alfa Aesar. Ammonium persulfate (APS; catalog no. AB00112), *N,N,N′,N′*-tetramethylethylenediamine (TEMED; catalog no. AB02020), tris [hyroxymethyl] aminomethane (Tris; catalog no. AB02000), and 20% sodium dodecyl sulfate solution in water (SDS; AB01922) were purchased from American Bio. Sodium chloride (NaCl; catalog no. 3624-01) was purchased from J.T. Baker.

### pan-ExM gelation chamber

The gelation chamber was constructed using a glass microscope slide (Sigma-Aldrich, catalog no. S8400) and two spacers, each consisting of a stack of two no. 1.5 22 × 22 mm coverslips (Fisher Scientific, catalog no. 12-541B), were glued with superglue to the microscope slide on both sides of the cell-adhered coverslip, with the cell-adhered coverslip glued in between. A no. 1.5 22 × 22 mm coverslip was used as a lid after adding the gel solution. This geometry yielded an initial gel thickness size of ~170 µm.

### First-round of expansion

HeLa and U-2OS cells, previously fixed as described in the Cell fixation section, were incubated in post-fix solution (0.7% FA + 1% AAm (w/v) in 1× PBS) for 6–7 h at 37 °C. Next, the cells were washed twice with 1× PBS for 10 min each on a rocking platform and embedded in the first expansion gel solution (19% (w/v) SA + 10% AAm (w/v) + 0.1% (w/v) DHEBA + 0.25% (v/v) TEMED + 0.25% (w/v) APS in 1× PBS). Gelation proceeded first for 15 min at room temperature (RT) and then 1.5 h at 37 °C in a humidified chamber. Coverslips with hydrogels were then incubated in ~1 mL denaturation buffer (200 mM SDS + 200 mM NaCl + 50 mM Tris in MilliQ water, pH 6.8) in 35 mm dishes for 15 min at RT. Gels were then transferred into denaturation buffer-filled 1.5 mL Eppendorf tubes and incubated at 73 °C for 1 h. Next, the gels were placed in petri dishes filled with MilliQ water for the first expansion. Water was exchanged at least twice every 1 h and then the gels were incubated overnight in MilliQ water. Gels expanded between 3.8× and 4.5× according to SA purity (see Reagents).

### Re-embedding in neutral hydrogel

Expanded hydrogels were incubated in a fresh re-embedding neutral gel solution (10% (w/v) AAm + 0.05% (w/v) DHEBA + 0.05% (v/v) TEMED + 0.05% (w/v) APS in 1× PBS) three times for 20 min each on a rocking platform at RT. Immediately after, residual gel solution was removed by extensive but gentle pressing with Kimwipes. The gels were then sandwiched between two pieces of no. 1.5 coverslips and incubated at 37 °C for 1.5 h in a nitrogen-filled humidified chamber. Next, the gels were detached from the coverslip and washed three times with 1× PBS for 30 min each on a rocking platform at RT. Gels were incubated in post-fix solution (0.7% FA + 1% (w/v) AAm in 1× PBS) for 15 min at RT and then for 6–9 h at 37 °C. The gels were subsequently washed three times with 1× PBS for 30 min each on a rocking platform at RT.

### Second-round of expansion

Re-embedded hydrogels were incubated in a fresh second hydrogel gel solution (19% (w/v) SA + 10% AAm (w/v) + 0.1% (w/v) BIS + 0.05% (v/v) TEMED + 0.05% (w/v) APS in 1× PBS) four times for 15 min each on a rocking platform on ice. Immediately after, residual gel solution was removed by extensive but gentle pressing with Kimwipes. The gels were then sandwiched between two pieces of no. 1.5 coverslips and incubated at 37 °C for 2 h in a humidified nitrogen-filled chamber. To dissolve DHEBA, gels were incubated in 0.2 M NaOH for 1 h on a rocking platform at RT. Gels were next detached from the coverslip and washed three times with 1× PBS for 30 min each on a rocking platform at RT. Subsequently, the gels were labeled with antibodies and pan-stained with NHS ester dyes. Finally, the gels were placed in petri dishes filled with MilliQ water for the second expansion. Water was exchanged at least twice every 1 h at RT, and then the gels were incubated overnight in MilliQ water. Gels expanded between 3.8× and 4.0× according to SA purity (see Reagents) for a final expansion factor of 13× to 20×. Note that for the ER images shown in Fig. 6b–e, the cleavable crosslinker BAC was used instead of BIS at a concentration of 0.1% (w/v).

### Antibody labeling post-expansion

For microtubule samples, gels were incubated for 24 h with monoclonal mouse anti-ɑ-tubulin antibody (DM1α; Sigma-Aldrich, catalog no. T6199) diluted to 1:250 in antibody dilution buffer (2% (w/v) BSA in 1× PBS). For mitochondria samples, gels were incubated for 24 h with rabbit anti-TOM20 antibody (Abcam, catalog no. ab78547) diluted to 1:250 in antibody dilution buffer. For centriole samples, gels were incubated for 24 h with rabbit polyclonal anti-polyglutamate chain (PolyE) antibody (Adipogen, catalog no. AG-25B-0030-C050) in antibody dilution buffer. For both Golgi and ER samples, gels were incubated for 36–40 h with polyclonal rabbit anti-GFP antibody (Invitrogen, catalog no. A11122) diluted to 1:250 in antibody dilution buffer. All primary antibody incubations were performed on a rocking platform at RT. Gels were then washed in PBS-T (0.1% (v/v)Tween 20 in 1× PBS) three times for 20 min each on a rocking platform at RT. Next, microtubule samples were incubated for 24 h with ATTO647N-conjugated anti-mouse antibodies (Sigma-Aldrich, catalog no. 50185) diluted to 1:250 in antibody dilution buffer, while mitochondria, ER, Golgi, and centriole samples were incubated for 24 h at RT with ATTO647N-conjugated anti-rabbit antibodies (Sigma-Aldrich, catalog no. 40839) diluted to 1:250 in antibody dilution buffer. All secondary antibody incubations were performed on a rocking platform at RT. The gels were subsequently washed in PBS-T three times for 20 min each, rinsed one time with 1× PBS, and stored in PBS at RT until subsequent treatments. Note that bovine serum albumin (BSA; catalog no. 001-000-162) was purchased from Jackson ImmunoResearch and Tween 20 (catalog no. P7949) was ordered from Sigma-Aldrich.

### NHS ester pan-staining post-expansion

After antibody labeling, gels were incubated for 1.5 h with either 20 µg/mL NHS ester-ATTO594 (Sigma-Aldrich, catalog no. 08741), 20 µg/mL NHS ester-ATTO532 (Sigma-Aldrich, catalog no. 88793) or 200 µM NHS ester-DY634 (Dyomics, catalog no. 634-01 A), dissolved in 100 mM sodium bicarbonate solution (Sigma-Aldrich, catalog no. SLBX3650) on a rocking platform at RT. The gels were subsequently washed three to five times in either 1× PBS or PBS-T for 20 min each on a rocking platform at RT. Note that for the experiment where we compared HeLa cells expanded never, once, or twice (Fig. 1), the same concentration of NHS ester-ATTO594 and labeling conditions were used.

### Palmitate pan-staining

Live 80%-confluent HeLa cells were incubated with 50 µM azide-functionalized palmitate (Thermofisher, catalog no. C10265) diluted in delipidated medium (DMEM + 10% charcoal-stripped FBS; Thermofisher, catalog no. A3382101) for 5 h at 37 °C and 5% CO_2_. Next, the cells were fixed with 3% FA + 0.1% GA in 1× PBS for 15 min at RT and processed according to pan-ExM protocols. Prior to NHS ester staining, CuAAC (Copper(I)-catalyzed Azide-Alkyne Cycloaddition) was performed using the Click-iT Protein Reaction Buffer Kit (Thermo Fisher, catalog no. C10276) according to manufacturer instructions. Alkyne-functionalized ATTO590 dye (Sigma-Aldrich, catalog no. 93990) was used at a concentration of 5 µM. After CuAAC, the gels were washed three times with 2% (w/v) delipidated BSA (Sigma Aldrich, catalog no. A4612) in 1× PBS for 20 min each on a rocking platform at RT.

### Maleimide pan-staining post-expansion

pan-ExM processed gels were reduced with 50 mM Tris(2-carboxyethyl)phosphine hydrochloride solution (TCEP) (Sigma-Aldrich, catalog no. 646547) in 1× PBS for 30 min at RT and subsequently incubated for 1.5 h in an inert environment with 20 µg/mL maleimide-ATTO594 (Sigma-Aldrich, catalog no. 08717) dissolved in deoxygenated 150 mM Tris-Cl pH 7.4 solution. The gels were then washed three times in either 1× PBS or PBS-T for 20 min each on a rocking platform at RT.

### SYTOX Green staining post-expansion

pan-ExM processed gels were incubated with SYTOX Green (Invitrogen, catalog no. S7020) diluted 1:3,000 in calcium- and magnesium-free HBSS buffer (Gibco, catalog no. 14170112) for 30 min on a rocking platform at RT. The gels were then washed three times with PBS-T for 20 min each on a rocking platform at RT.

### pan-ExM sample mounting

After expansion, the gels were mounted on Poly-L-Lysine-coated glass-bottom dishes (35 mm; no. 1.5; MatTek). A clean 18-millimeter diameter Poly-L-Lysine-coated coverslip (Marienfeld, catalog no. 0117580) was put on top of the gels after draining excess water using Kimwipes. The samples were then sealed with a two-component silicone glue (Picodent Twinsil, Picodent, Wipperfürth, Germany). After the silicone mold hardened (typically 15–20 min), the samples were stored in the dark at RT until they were imaged. Note that gels imaged with an oil objective were incubated overnight in 30% glycerol (Teknova, catalog no. G1797) prior to mounting.

### Image acquisition

Confocal and STED images were acquired using a Leica SP8 STED 3X equipped with a SuperK Extreme EXW-12 (NKT Photonics) pulsed white light laser as an excitation source and a Onefive Katana-08HP pulsed laser as depletion light source (775-nm wavelength). All images were acquired using either a HC PL APO 63×/1.2 water objective, HC PL APO 86×/1.2 water CS2 objective, HC PL APO 63×/1.40-0.60 oil objective, or a HC PL APO 100×/1.40 NA oil immersion CS2 objective. Application Suite X software (LAS X; Leica Microsystems) was used to control imaging parameters. ATTO532 was imaged with 532-nm excitation. ATTO594 was imaged with 585-nm excitation and 775-nm depletion wavelengths. ATTO647N was imaged with 647-nm excitation and 775-nm depletion wavelengths. DY634 was imaged with 634-nm excitation. SYTOX Green and MitoTracker Orange were excited by 488-nm and 555-nm excitation light, respectively.

Widefield images to measure protein retention were obtained with a Leica tissue culture microscope (DM IL LED FLUO) equipped with a 10×/0.22 NA air objective. An Andor Clara CCD camera operated by MicroManager was used to record images.

### Protein retention assay

HeLa cells were transfected with either GFP-ManII or GFP-Sec61β and plated at 75,000 cells per 12-mm fibronectin-coated glass coverslip. Non-expanded cells from the same experiment were stored in 1× PBS at 4 °C after fixation and were permeabilized with 0.1% Triton X-100 (Sigma-Aldrich, catalog no. T8787) in 1× PBS prior to antibody labeling. All non-expanded, once- and twice-expanded samples were subjected to the same antibody labeling scheme described in Antibody labeling post-expansion. However, ATTO594-conjugated anti-rabbit antibodies (1:250; Sigma-Aldrich, catalog no. 77671) were used instead of ATTO647N-conjugated antibodies. Additionally, samples were stained with SYTOX Green as described in SYTOX Green staining post-expansion. All samples were imaged in deionized water with a widefield Leica tissue culture microscope (DM IL LED FLUO) using a 10×/0.22 NA air objective (see Image Acquisition) and the same LED light intensity.

To measure protein retention in the first expansion step, we compared the total fluorescence signal (TFS) of ATTO594 between non-expanded and once expanded cells expressing GFP-Sec61β and immunolabeled against GFP. Since the ER spreads throughout the whole cytoplasm, TFS for these samples was quantified by measuring the total background-corrected mean fluorescence signal per field of view and dividing it by the number of cells in the field of view. Cells were counted based on the SYTOX Green nuclear staining using FIJI’s 3D Objects counter. Cell numbers were manually corrected for nuclei which were so close that the automatic segmentation merged them into single objects (typically the case for 5–20% of the nuclei). Background levels were determined by averaging the signal determined in a manually selected area containing no cells. 5 fields of view containing between 419 and 653 cells each were analyzed for non-expanded samples; 10 fields of view containing 21–58 cells each were analyzed for samples expanded once.

Because signal-to-background levels dropped to values too low to provide reliable results using the described method for the ER staining in samples expanded twice, we chose a different approach using more localized labeling of the Golgi complex for these samples. To measure protein retention in the second expansion step, we compared the TFS of ATTO594 between once-expanded and twice-expanded cells expressing GFP-ManII and immunolabeled against GFP. TFS for these samples was quantified by multiplying the manually-identified total area occupied by stained Golgi stacks in every cell with the background-corrected mean fluorescence signal in that area. Background levels were determined by averaging the signal determined in a manually selected area within a cell containing no GFP-ManII signal. 60 cells in 10 fields of view were analyzed for samples expanded once and 67 cells in 45 fields of view were analyzed for samples expanded twice. For all measurements, TFS was corrected for the different camera exposure times used. Images were processed using FIJI/ImageJ software. Results are summarized in Supplementary Fig. 18.

### Image processing

Images were visualized, smoothed, and contrast-adjusted using FIJI/ImageJ or Imspector software. STED and confocal images were smoothed for display with a 0.5 to 1-pixel sigma Gaussian blur. Minimum and maximum brightness were adjusted linearly for optimal contrast. The TOM20 data set (Fig. 2d–f) was corrected for bleedthrough of the NHS ester channel by subtracting a constant fraction of the latter from the former using Imspector. The confocal ManII (Fig. 7) and mitotic cell NHS ester (Supplementary Fig. 9) data sets were corrected for bleedthrough of the NHS ester and SYTOX Green channels respectively by subtracting a constant fraction of the latter from the former using the Image Expression Parser tool in FIJI.

Cristae and Golgi inter-cisternal distance measurements were performed using FIJI. 10-pixel thick line profiles were taken approximately perpendicular to the cristae and Golgi stack orientations and peak-to-peak distances were extracted from the profiles. For mitochondrial cristae, 123 line profiles were drawn from 4 independent experiments. For Golgi inter-cisternal distance measurements, 193 line profiles were drawn from 3 independent experiments. The diameter of ER tubules was determined in FIJI using the Point Tool by manually measuring two positions arranged perpendicular to the orientation of clearly discernible tubules and located at the crests of the signal denoting each side of the tubule. The Euclidean distance between them was used as a measure of the ER tubule diameter. For this measurement, 142 ER tubule widths were extracted from 2 cells in 1 sample. Results are summarized in Fig. 6i–k.

All line profiles were extracted from the images using the Plot Profile tool in FIJI/ImageJ.

### Expansion factor calculation

Images of HeLa cell nuclei in non-expanded and pan-ExM expanded samples stained with SYTOX Green (1:3,000) were acquired with a Leica SP8 STED 3X microscope using a HCX PL Fluotar 10×/0.30 dry objective. Average nuclear cross-sectional areas were determined using FIJI/ImageJ software. To calculate the expansion factor, the average nuclear cross-sectional area in pan-ExM samples was divided by the average nuclear cross-sectional area of non-expanded samples. The square root of this ratio represents an estimate of the linear expansion factor. Results are summarized in Supplementary Fig. 11c.

### Expansion homogeneity calculation

To compare nuclei, mitochondria, and microtubules pre- and post-expansion, U-2OS cells were cultured as specified in Cell culture and live-labeled with MitoTracker Orange as described in MitoTracker Orange staining. After fixation with 3% FA + 0.1% GA in 1× PBS for 5 min at RT, cells were incubated in post-fix solution (0.7% FA + 1% (w/v) AAm in 1× PBS) for 7 h at 37 °C, permeabilized with 0.1% Triton X-100 in 1× PBS for 5 min on a rocking platform at RT, and labeled with a mouse monoclonal anti-ɑ-tubulin antibody (MT antibody 1; Sigma-Aldrich, catalog no. T5168) diluted in cell antibody dilution buffer (1% (w/v) BSA + 0.2% TX-100 in 1× PBS) for 1 h on a rocking platform at RT. They were then washed three times with wash buffer (0.05% TX-100 in 1× PBS) for 5 min each, labeled with ATTO647N-conjugated anti-mouse antibodies (Sigma-Aldrich, catalog no. 50185) diluted to 1:1,000 in cell antibody dilution buffer for 1 h on a rocking platform at RT, and stained with Hoechst (abcam, catalog no. ab228551) diluted to 1:10,000 in 1× PBS for 20 min on a rocking platform at RT. The samples were next washed three times with wash buffer for 5 min each and rinsed with 1× PBS.

Pre-expansion image stacks of nuclei (Hoechst), mitochondria (MitoTracker Orange), and microtubules (MT antibody 1) were acquired. The cells were then immediately processed with pan-ExM protocol. For post-expansion microtubule labeling, gels were labeled with a different mouse monoclonal anti-ɑ-tubulin antibody (MT antibody 2; Sigma-Aldrich, catalog no. T6199) diluted 1:250 in antibody dilution buffer (2% (w/v) BSA in 1× PBS) for 6 h at 37 °C and 6 h on a rocking platform at RT, washed three times with PBS-T for 20 min each, and labeled with the same ATTO647N-conjugated anti-mouse antibodies as above. Gels were then stained with SYTOX Green dye diluted 1:3,000 in calcium- and magnesium-free HBSS buffer for 45 min on a rocking platform at RT, washed three times with PBS-T for 30 min each at 37 °C, and expanded in MilliQ water as described above. Post-expansion image stacks of nuclei (SYTOX Green), mitochondria (MitoTracker Orange), and microtubules (MT antibody 2) were acquired. Maximum projection images of corresponding pre- and post-expansion image stacks were generated with the FIJI/ImageJ z projection tool. Additionally, post-expansion images of mitochondria and microtubules were despeckled and masks were created manually to exclude regions of no features for microtubule samples.

To determine spatial sample distortion, post-expansion images were smoothed with a 2-pixel Gaussian blur and first registered to the pre-expansion image of the same field of view with either a similarity transform (uniform scaling, rotation, and translation) or an affine transform (scaling, shear, rotation, and translation). FIJI TurboReg plugin was used for this initial registration. The similarity- and affine-registered post-expansion images were registered again to the pre-expansion images with a B-spline-based non-rigid registration package in Matlab^8^. The similarity measure (error) was set to squared pixel distance and the penalty of registration was set to 1e-1 (nuclei images) or 1e-2 (mitochondria and microtubule images). Using the deformation vector field from the output B-spline transformation parameters, the root mean square (RMS) error of expansion was calculated across different distance measurements. The deformation field was applied to the coordinates of either a binary outline of the pre-expansion image (nuclei images) or its binary skeleton (mitochondria and microtubule images). The distance between every two pairs of points in the pre-expansion image binary image (*d*_*i*) and the corresponding deformed coordinates (*d*_def) were calculated. The RMS error is the absolute difference of these distance measurements (RMS = abs(*d*_def - *d*_*i*)). RMS error was calculated for every combination of points across distances of 20 µm (nuclei images), 10 µm (mitochondria images), and 8 µm (microtubule images). For nuclei, 5 cells in 5 fields of view were analyzed. For mitochondria, 14 fields of view in 5 cells were analyzed. For microtubules, 5 fields of view in 4 cells were analyzed. Results are summarized in Supplementary Fig. 12.

To quantify the expansion factor of nuclei, mitochondria, and microtubules. Similarity-registered post-expansion images and the corresponding pre-expansion images were cropped using four manually-identified landmark features in both images. To determine the expansion factor, the area of the cropped post-expansion image was divided by the area of the cropped pre-expansion image. The square root of this ratio represents the linear expansion factor. Results are summarized in Supplementary Fig. 11.

### pan-ExM modified protocol

To test whether the mechanism of the second expansion is primarily polymer entanglement or chemical crosslinking between the sample and the final expansion hydrogel, a modified pan-ExM protocol was developed. After denaturation (Step 3, Supplementary Fig. 1), possible reactive Schiff base groups on proteins that may react covalently with acrylamide monomers were quenched by incubating the gels in 0.1% sodium borohydride (Sigma-Aldrich, catalog no. 452882) in 1× PBS for 7 min at RT followed by an incubation with 100 mM glycine (Sigma-Aldrich, catalog no. G8898) in 1× PBS for 10 min at RT. Additionally, the second post-fixation step (Step 7, Supplementary Fig. 1) was omitted to prevent crosslinking of the sample to the second expansion hydrogel. The remaining steps are identical to the original protocol.

### Centriole roundness quantification

Image stacks of PolyE-labeled U-2OS mature centrioles were acquired by confocal microscopy and axial-view centriole image stacks were selected for quantification. Volume Viewer plugin in FIJI was used to align image stacks that were not perfectly axial. Manually selected images of the distal region were smoothed with a 1-pixel Gaussian blur and converted to binary using automatic thresholding in FIJI. Using the shape descriptor tool in FIJI, centriole roundness was measured for both post-fixed centrioles (standard protocol; *n* = 7 centrioles) and quenched/no post-fix centrioles (pan-ExM modified protocol; *n* = 8 centrioles). Results are summarized in Supplementary Fig. 15.

### Centriole length-to-width quantification

Image stacks of PolyE-labeled and NHS ester pan-stained mature centrioles were acquired by confocal microscopy and lateral view image stacks were selected for quantification. Similarly as above, Volume Viewer plugin in FIJI was used to align image stacks that were not perfectly lateral. To compare the length-to-width ratio of centrioles, 5-pixel thick line profiles of NHS ester pan-staining along the length of the centrioles were drawn to measure centriolar length, and 5-pixel thick line profiles of PolyE staining along the width of the centriole were drawn to measure centriolar width.The peak-to-peak distances were extracted from these profiles and the ratio is the centriole length-to-width ratio^12^. This ratio was measured for both post-fixed centrioles (standard protocol; *n* = 17 centrioles) and quenched/no post-fix centrioles (pan-ExM modified protocol; *n* = 22 centrioles). Results are summarized in Supplementary Fig. 15.

### Measurement of antibody labeling efficiency

To test whether the treatments required to dissolve several common cleavable crosslinkers (Supplementary Fig. 27) play a role in reducing post-expansion antibody labeling efficiency, four U-2OS cell samples live-labeled with MitoTracker Orange as described in MitoTracker Orange staining were expanded once (Steps 1–5, Supplementary Fig. 1) using a hydrogel prepared with 0.1% (w/v) BIS (instead of 0.1% (w/v) DHEBA). After the denaturation step (Step 4, Supplementary Fig. 1), one gel was expanded (CTRL) right after. The second gel was treated with 0.2 M NaOH for 1 h at RT (Treatment 1) and then expanded. The third gel was treated with 25 mM sodium periodate (Sigma-Aldrich, catalog no. 311448) diluted in 100 mM sodium acetate buffer (Sigma-Aldrich, catalog no. S7899), adjusted to pH 6.0, for 1 h at RT (Treatment 2) and then expanded. Finally, the fourth gel was treated with 0.25 M TCEP diluted in 1 M Tris-Cl buffer, adjusted to pH 7.5, for 18 h at RT (Treatment 3) and then expanded. Note that we verified that Treatment 1 can dissolve a gel composed of 19% (w/v) SA + 10% (w/v) AAm + 0.1% (w/v) DHEBA + 0.25% (w/v) APS + 0.25% (v/v) TEMED in 1 h at RT; Treatment 2 can dissolve a gel composed of 19% (w/v) SA + 10% (w/v) AAm + 0.2% (w/v) DATD + 0.25% (w/v) APS + 0.25% (v/v) TEMED in 1 h at RT; and Treatment 3 can dissolve a gel composed of 19% (w/v) SA + 10% (w/v) AAm + 0.1% (w/v) BAC + 0.25% (w/v) APS + 0.25% (v/v) TEMED in 18 h at RT. After expansion, all four gels were immunolabeled with both a mouse monoclonal anti-ɑ-tubulin antibody (1:500; Sigma-Aldrich, catalog no. T5168) and a rabbit polyclonal anti-TOM20 antibody (1:500; Abcam, catalog no. ab78547) diluted in antibody dilution buffer (2% (w/v) BSA in 1× PBS) for 12 h on a rocking platform at RT. The gels were washed three times with PBS-T for 20 min each and then immunolabeled with both ATTO647N-conjugated anti-mouse antibodies (1:500; Sigma-Aldrich, catalog no. 50185) and ATTO594-conjugated anti-rabbit antibodies (1:500, Sigma-Aldrich, catalog no. 77671) diluted in antibody dilution buffer for 6 h on a rocking platform at RT. The gels were washed with PBS-T for 20 min each on a rocking platform at RT and expanded again in MilliQ water to the final expansion factor of ~4.5. 3-color images of microtubules (anti-ɑ-tubulin) and mitochondria (MitoTracker Orange and anti-TOM20) were acquired for each condition.

To measure TOM20 signal, background-corrected total fluorescence signal was calculated for each ROI containing mitochondria. This value was divided by the area occupied by mitochondria as calculated from the area of a mask generated from the corresponding MitoTracker Orange signal. Between 23 to 29 fields of view were quantified in 5 cells for every condition. To measure ɑ-tubulin signal, line profiles of microtubules were drawn from the images using the Plot Profile tool in FIJI/ImageJ and the background-corrected peak value was extracted. Between 104 and 123 profiles were drawn in 5 cells for every condition. Results are summarized in Supplementary Fig. 25.

### Statistics and reproducibility

For all quantitative experiments, the number of samples and independent reproductions are listed in the figure legends. An unpaired two-tailed *t*-test in Graphpad Prism 8 was used to analyze the data presented in Supplementary Figs. 15, 18 and 25.

### Reporting summary

Further information on research design is available in the Nature Research Reporting Summary linked to this Article.

## Supplementary information

Supplementary Information

Description of Additional Supplementary Files

Supplementary Movie 1

Supplementary Movie 2

Supplementary Movie 3

Supplementary Movie 4

Supplementary Movie 5

Reporting Summary

## Data Availability

The datasets generated and/or analyzed during the current study are available from the corresponding author on reasonable request.
