# Supplementary material for: Light microscopy of proteins in their ultrastructural context
Source: Nat Commun. 2020 Jul 31;11:3850. doi: 10.1038/s41467-020-17523-8 (PMC7395138; doi:10.1038/s41467-020-17523-8)
Supplement: Supplementary file 1 — Supplementary Information [file 41467_2020_17523_MOESM1_ESM.pdf]

Supplementary Information

Light microscopy of proteins in their ultrastructural context

M'Saad and Bewersdorf

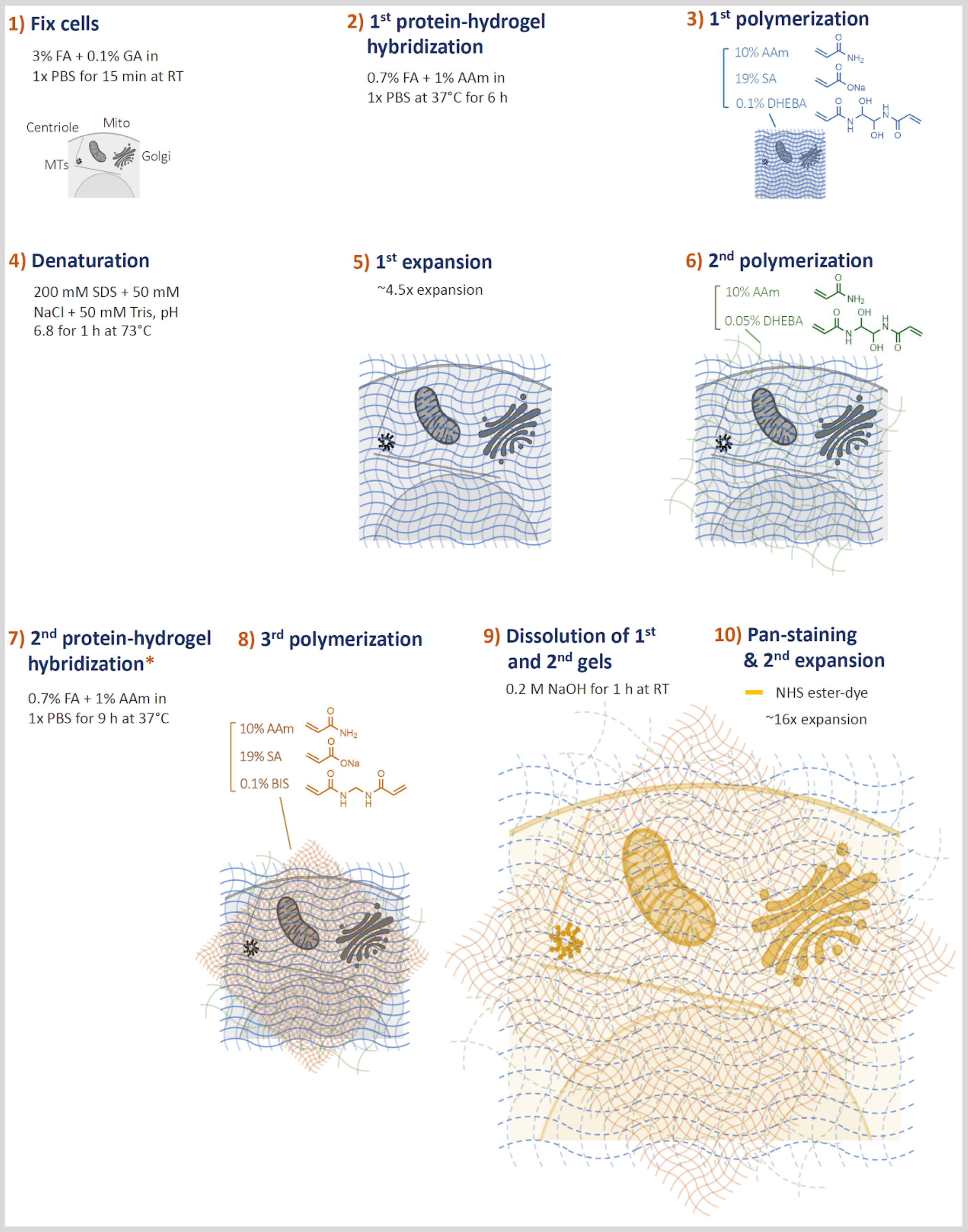

Supplementary Figure 1. Protocol schematic of pan-ExM. *abbreviations: FA: formaldehyde; GA: glutaraldehyde; AAm: acrylamide; SA: sodium acrylate; DHEBA: N,N'-(1,2-dihydroxyethylene)bis-acrylamide; BIS: N,N'-methylenebisacrylamide; SDS: sodium dodecyl sulfate. Note that all percent concentrations are mass per volume. Step 7, denoted by the orange asterisk, when omitted, does not significantly impact centriole roundedness and length-to-width diameter (pan-ExM modified protocol; see Methods and Supplementary Figure 15) and shows typical NHS ester pan-staining (Supplementary Figure 19). Clipart created with Biorender.com.*

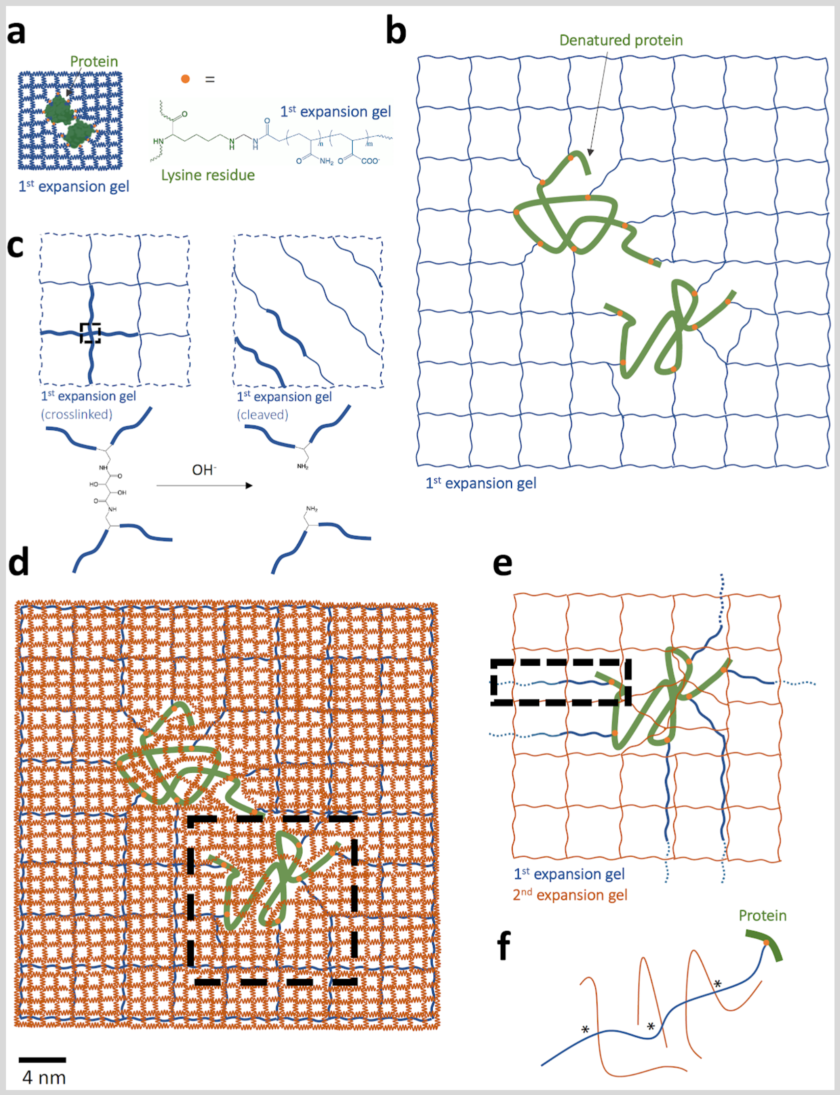

***Supplementary Figure 2. Proposed pan-ExM expansion mechanism. a**, schematic of two proteins (green) embedded in a first expansion gel (blue) before sample denaturation and expansion. Orange dot represents the chemical structure of protein-polymer crosslinks. **b**, schematic of proteins denatured (green) and expanded in the first expansion hydrogel (blue) **c**, schematic representing dissolution of the first expansion hydrogel: base hydrolysis of N,N'-(1,2-dihydroxyethylene)bisacrylamide (DHEBA) converts the crosslinked first expansion hydrogel network (black dashed box) into linear polymer chains. **d**, schematic of proteins (green) embedded in the second expansion gel (orange) after the first expansion and before the second expansion. The neutral polyacrylamide gel, in which the sample is additionally embedded, is not drawn for simplicity. **e**, schematic of a protein-polymer hybrid after the second expansion, shown in the black dashed box in d before the second expansion, entangled in the second expansion gel (orange) after dissolution of the first expansion gel (blue). **f**, schematic of the polymer network shown in the dashed black box in e illustrating the definition of polymer entanglement: a polymer chain is likely entangled if it crosses an arbitrary plane 3 times (asterisks). Clipart in a created with Biorender.com.*

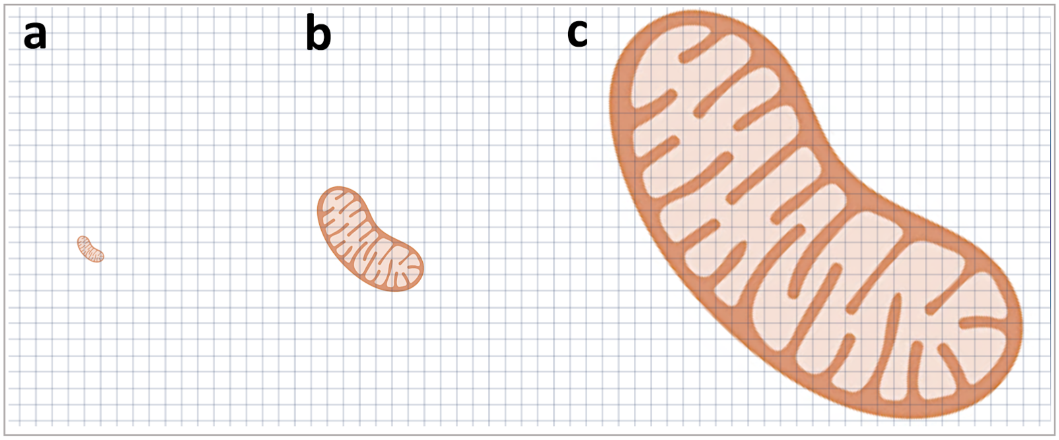

***Supplementary Figure 3. 16-fold iterative expansion drawn to scale. a**, schematic of a mitochondrion before sample expansion. **b**, schematic of a mitochondrion after 4-fold expansion. **c**, schematic of a mitochondrion after 16-fold expansion. Clipart created with Biorender.com.*

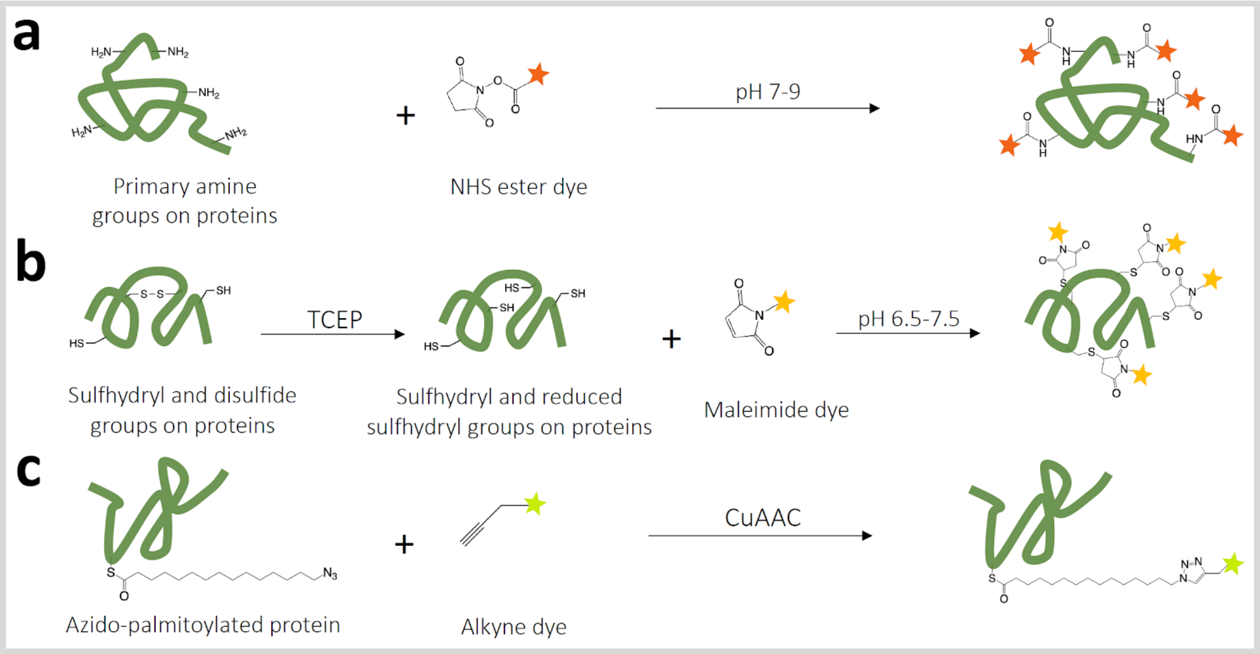

***Supplementary Figure 4. Examples of pan-stains. a**, reaction of primary amines (-NH2) on proteins (green) using N-Hydroxysuccinimide (NHS) ester-conjugated dyes (orange star) yields proteins with stable protein-dye amide linkages. **b**, reaction of sulfhydryl groups (-SH) on proteins (green) using maleimide-conjugated dyes (yellow star) after sample reduction with tris(2-carboxyethyl)phosphine (TCEP) yields proteins with stable protein-dye thioether linkages. **c**, copper-catalyzed azide/alkyne cycloaddition (CuAAC) reaction of azido palmitoylated proteins (green) with alkyne-conjugated dyes (green star) yields proteins with stable triazole linkages.*

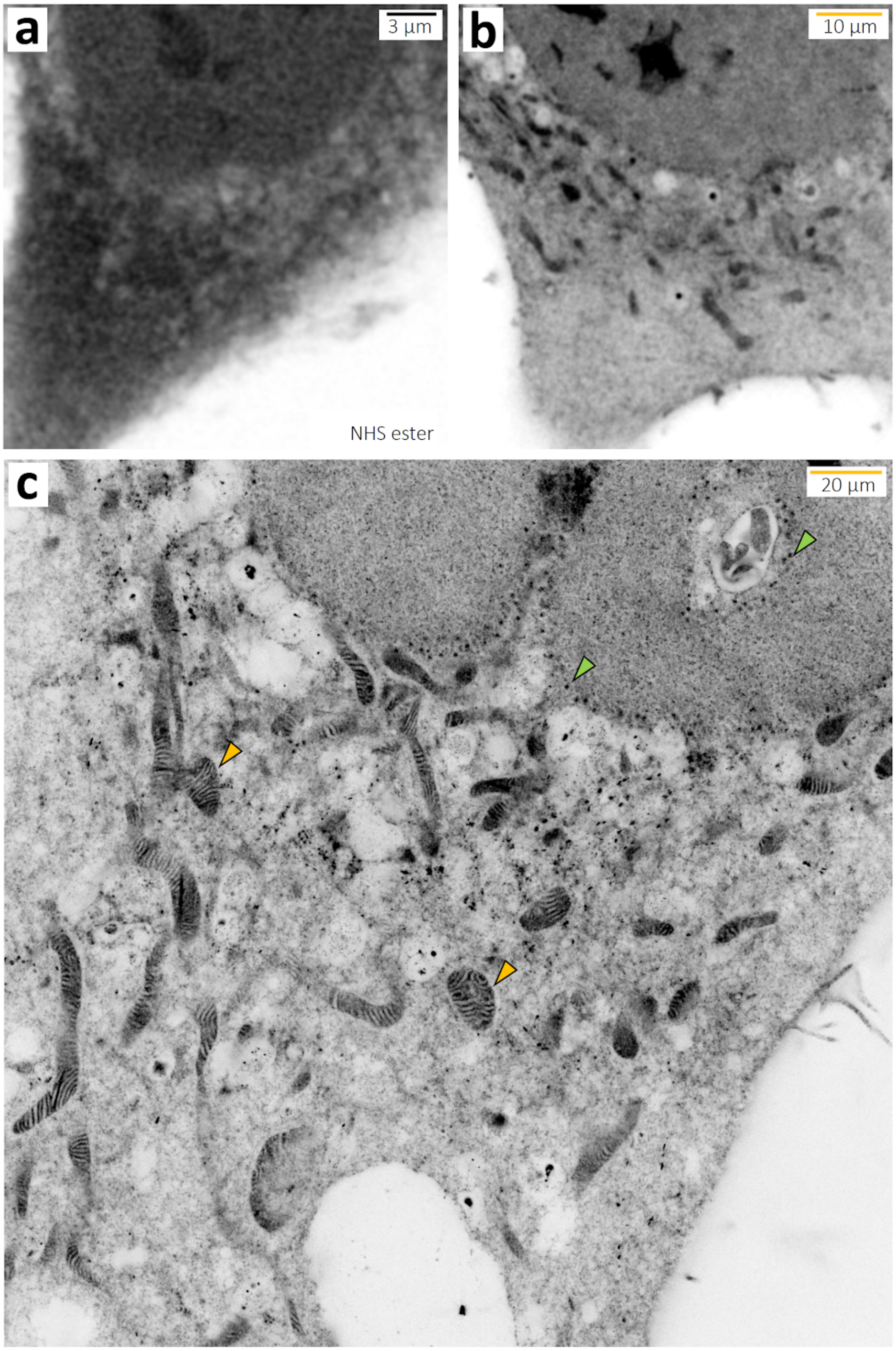

***Supplementary Figure 5. pan-ExM image of a HeLa cell pan-stained with NHS ester and displayed with an inverted color table resembling EM images. a**, non-expanded HeLa cell. **b**, HeLa cell expanded once. **c**, pan-ExM expanded HeLa cell revealing hallmark ultrastructural features such as mitochondria cristae (yellow arrowheads) and nuclear pore complexes (NPCs) (green arrowheads). Representative images from 3 (a,b) and 11 (c) independent experiments. All figures are displayed with a white-to-black color table. Yellow scale bars are not corrected for the respective expansion factor.*

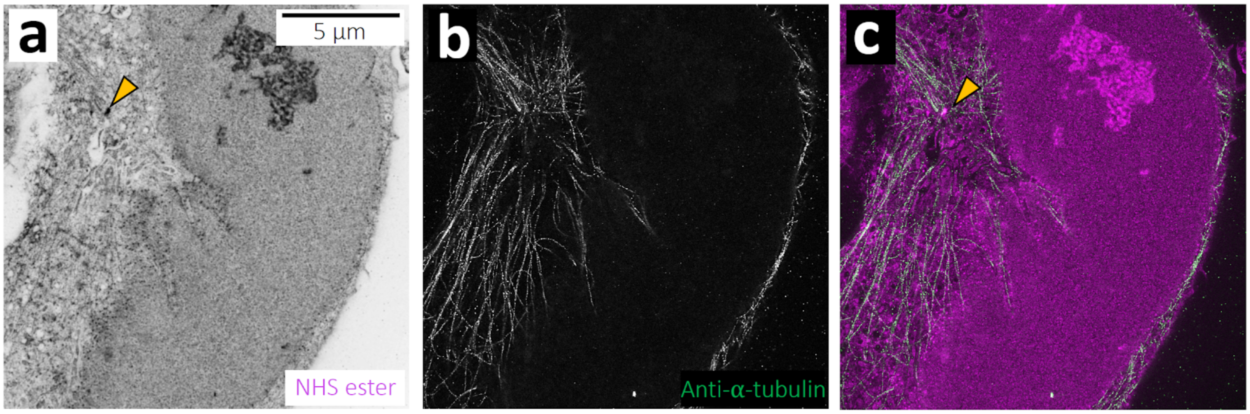

***Supplementary Figure 6. pan-ExM is compatible with microtubule labeling. a**, NHS ester pan-stained HeLa cell. The yellow arrowhead points at an amine-rich region corresponding to the centrosome. **b**, same area as in a, showing anti-α-tubulin immunostaining. **c**, overlay of a and b. The yellow arrowhead points at the centrosome. Representative images from 3 (a-c) independent experiments. Panel a is displayed with a white-to-black color table. Panel b is displayed with a black-to-white color table. The scale bar is corrected for the determined expansion factor.*

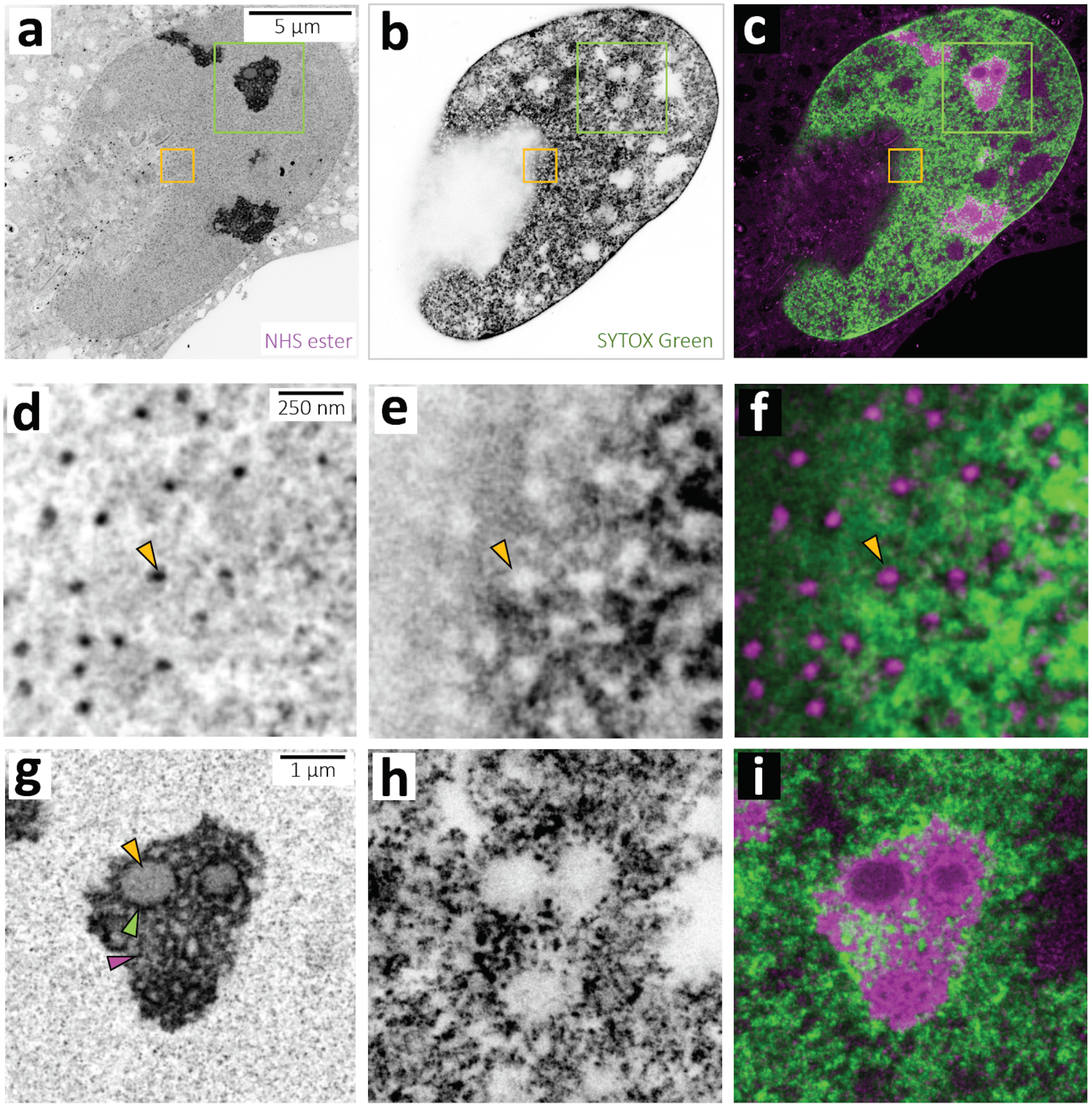

***Supplementary Figure 7. pan-ExM reveals nuclear architecture in a U-2OS cell in interphase. a**, NHS ester pan-stained U-2OS cell in interphase. **b**, SYTOX Green nucleic acid stain corresponding to area shown in a. **c**, overlay of a and b. **d**, magnified area in the yellow box in a showing amine-rich region corresponding to nuclear pore complexes (NPCs). The yellow arrowhead points at one NPC. **e**, magnified area in the yellow box in b showing chromatin and circular regions excluding chromatin which correspond to the areas occupied by NPCs in d. The yellow arrowhead points at one such region. **f**, overlay of d and e. **g**, magnified area in the green box in a showing ultrastructural details of a nucleolus. The yellow arrowhead points at the fibrillar center (FC). The green arrowhead points at the dense fibrillar component (DFC). The magenta arrowhead points at the granular component (GC). **h**, magnified area in the green box in b showing chromatin and regions excluding chromatin which correspond to the area occupied by the nucleolus in g. **i**, overlay of g and h. Representative images from 5 (a-i) independent experiments. Panels a,b,d,e,g,h are displayed with a white-to-black color table. All scale bars are corrected for the determined expansion factor.*

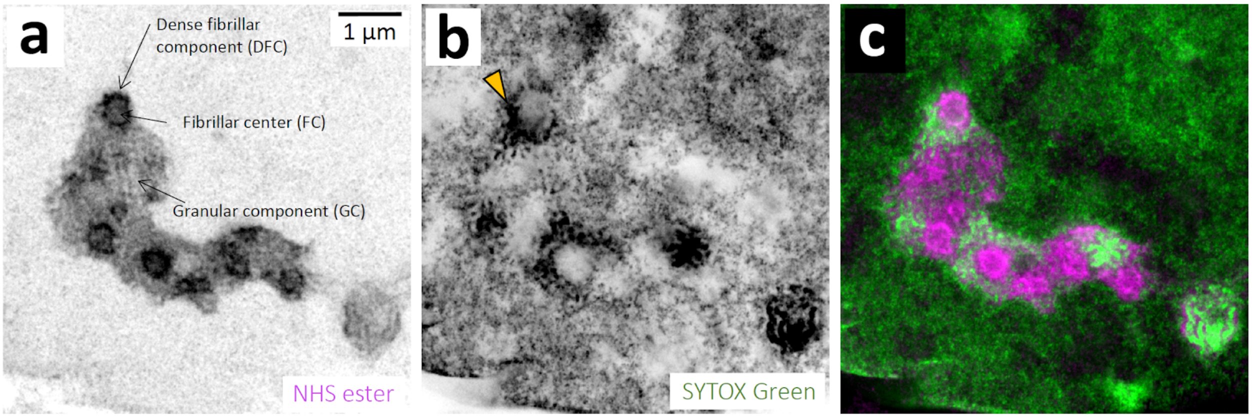

***Supplementary Figure 8. pan-ExM reveals nucleoli ultrastructure in HeLa cells. a**, NHS ester rich area in a HeLa cell nucleus representing a nucleolus. Annotations point at the dense fibrillar component (DFC), fibrillar center (FC), and granular component (GC). **b**, SYTOX Green DNA stain corresponding to the same field of view shown in a. The yellow arrowhead points at a nucleic acid-rich region surrounding a dense fibrillar center (DFC). **c**, overlay of a and b. Representative images from at least 3 independent experiments. Panels a and b are displayed with a white-to-black color table. The scale bar is corrected for the determined expansion factor.*

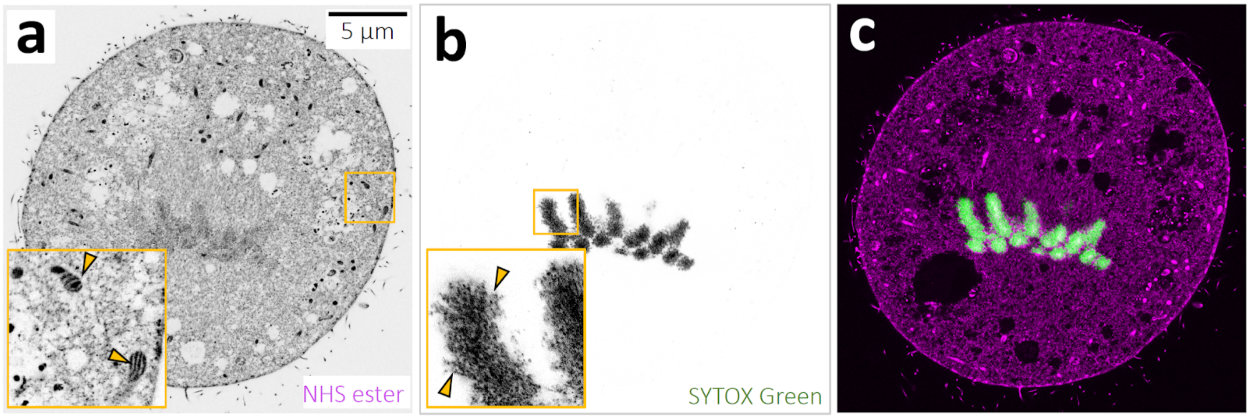

***Supplementary Figure 9. pan-ExM reveals mitotic HeLa cell ultrastructure. a**, NHS ester pan-stained mitotic HeLa cell. The inset shows the zoomed-in yellow box and reveals densely labeled mitochondria (yellow arrowheads). **b**, SYTOX Green stain corresponding to the field of view shown in a showing chromosomes. The inset shows the zoomed-in yellow box and reveals the polymer brush-like architecture model of mitotic chromosomes (yellow arrowheads). **c**, overlay of a and b. Representative images from at least 3 (a-c) independent experiments. Panel a is corrected for crosstalk (see Methods for details). Panels a and b are displayed with a white-to-black color table. The scale bar is corrected for the determined expansion factor.*

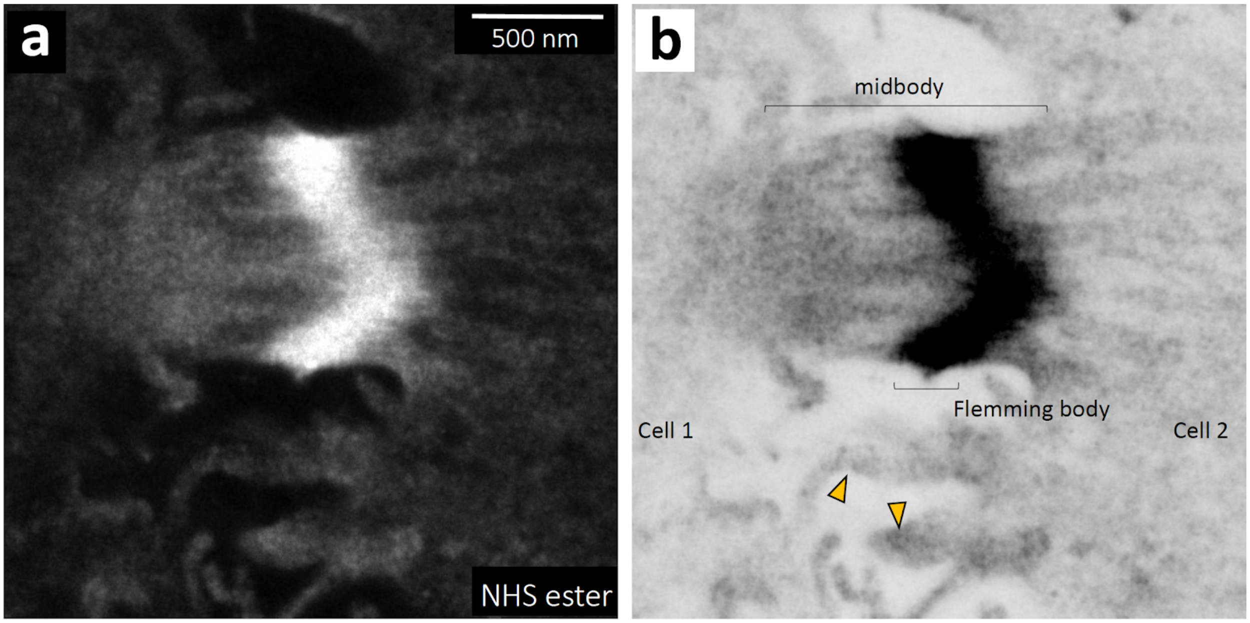

***Supplementary Figure 10. pan-ExM reveals cleavage furrow ultrastructure in a dividing U-2OS cell. a**, NHS ester pan-stained cleavage furrow. **b**, annotated image in a with inverted color table depicting the midbody and amine-rich Flemming body. The yellow arrowheads point at protrusions of the plasma membrane. Representative image from 1 (a,b) independent experiment. Panel a is displayed with a black-to-white color table. Panel b is displayed with a white-to-black color table. The scale bar is corrected for the determined expansion factor. The shown images were recorded with a standard confocal microscope.*

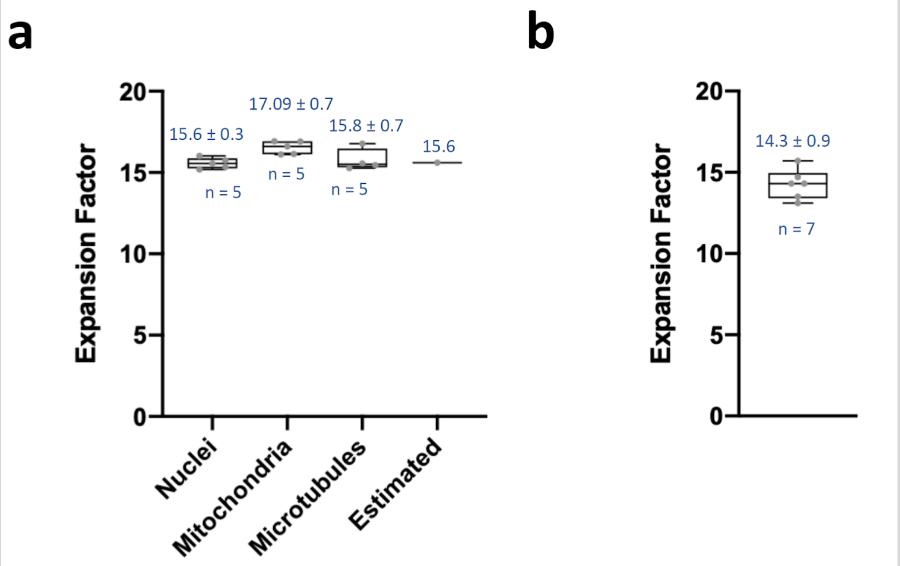

c

Experiment number	Expansion factor
Experiment 1	13.5
Experiment 2	14.3
Experiment 3*	21.0
Experiment 4	14.7
Experiment 5	13.1
Experiment 6	14.3
Experiment 7	15.7

Supplementary Figure 11. pan-ExM expansion factor calculation across different structures. a*, Expansion factor determined from registering pre-expansion to post-expansion images of nuclei, mitochondria, and microtubules (Nuclei: n = 5 cells; Mitochondria: n = 5 cells; Microtubules: n = 5 cells, N = 1 experiment). The figure also shows the estimated expansion factor determined from averaged nuclei cross-section measurements in the same experiment (n = 39 nuclei; N = 1 experiment; see Methods for quantification details).* ***b****, average expansion factor achieved with pan-ExM (n = 6 experiments).* ***c****, table showing expansion factors calculated from averaged nuclei cross-section measurements from 7 independent experiments. In Experiment 3, the crosslinker N,N'-cystaminebisacrylamide (BAC) was used at 0.1% (w/v) concentration in the final hydrogel instead of N,N'-methylenebisacrylamide (BIS). The expansion factor determined from Experiment 3 was not included in determining the average expansion factor (Panel b). Medians and interquartile ranges are shown with whiskers drawn down to the minimum and maximum values. Means ± standard deviations are reported.*

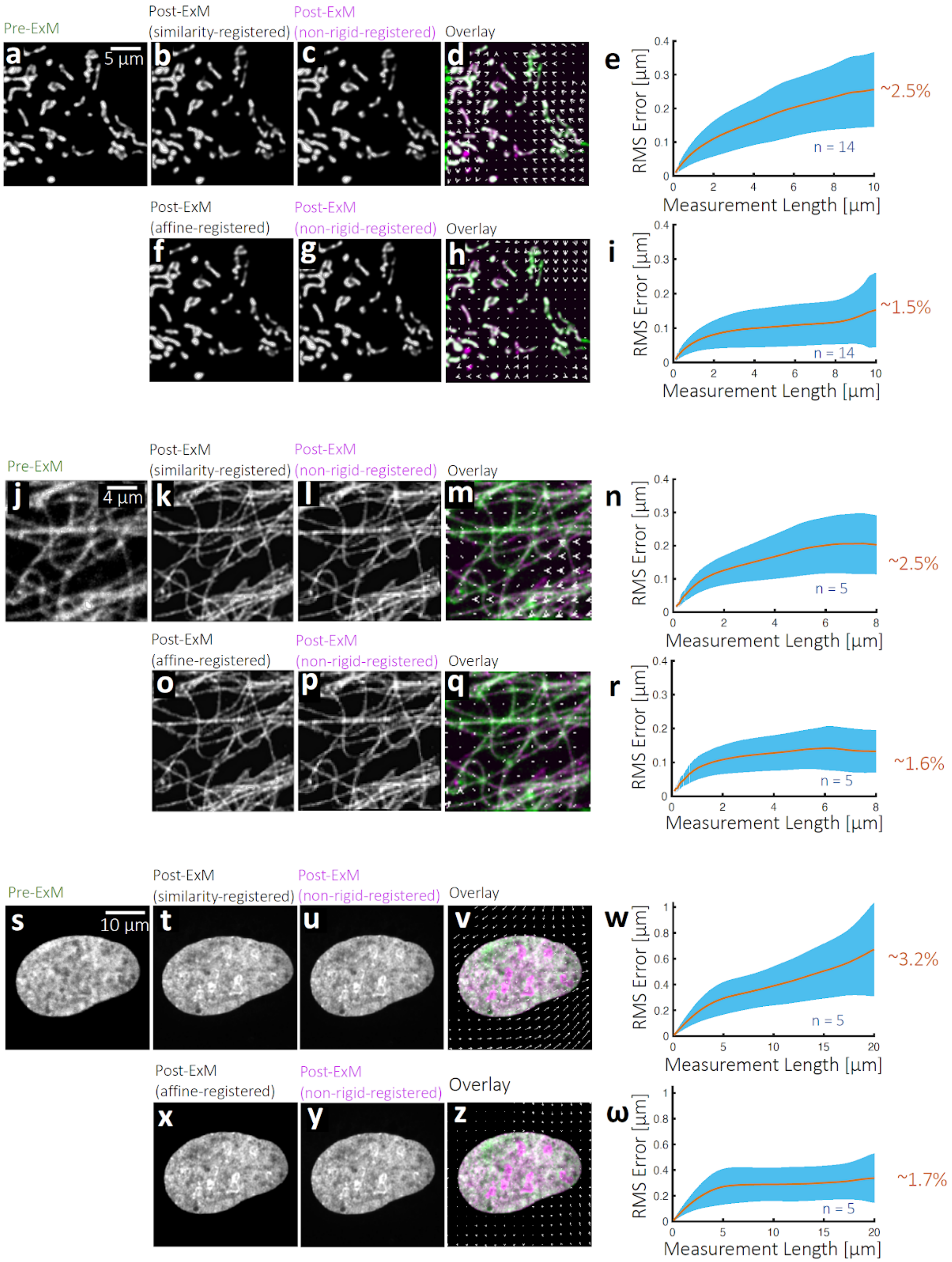

Pre-ExM
Post-ExM (similarity-registered)
Post-ExM (non-rigid-registered)
Overlay
Post-ExM (affine-registered)
5 µm
4 µm
10 µm
RMS Error [µm]
Measurement Length [µm]
~2.5%
~1.5%
~2.5%
~1.6%
~3.2%
~1.7%
n = 14
n = 5

Supplementary Figure 12. Sample deformation over length scales of several micrometers for mitochondria, microtubules, and cell nuclei. a-i*, evaluation of sample deformation in mitochondria.* ***a****, pre-expansion image of mitochondria. b, post-expansion image of the same field of view similarity-registered to image shown in a.* ***c****, post-expansion image of the same field of view non-rigid-registered to image shown in a.* ***d****, overlay of b and c with distortion vector field (white arrows).* ***e****, root-mean square (RMS) error quantification as a function of distance (n = 14 FOVs; N = 5 cells).* ***f****, post-expansion image of the same field of view affine-registered to image shown in a.* ***g****, post-expansion image of the same field of view non-rigid-registered to image shown in a.* ***h****, overlay of a and g with distortion vector field (white arrows).* ***i****, RMS error quantification as a function of distance (n = 14 FOVs; N = 5 cells).* ***j-r****, evaluation of sample deformation in mitochondria. Layout is analogous to that in a-i (n = 5 FOVs; N = 4 cells for both similarity and affine-registered data).* ***s-ω****, evaluation of sample deformation in nuclei. Layout is analogous to that in a-i (n = 5 FOVs; N = 5 cells for both similarity and affine-registered data). Representative images from 14 (a-d, f-h), 5 (j-m, o-q), and 5 (s-v, x-z) fields of view. In the plots e,I,n,r,w,ω, the orange line corresponds to the mean and blue error bars correspond to the standard deviation. See Methods for RMS error quantification details.*

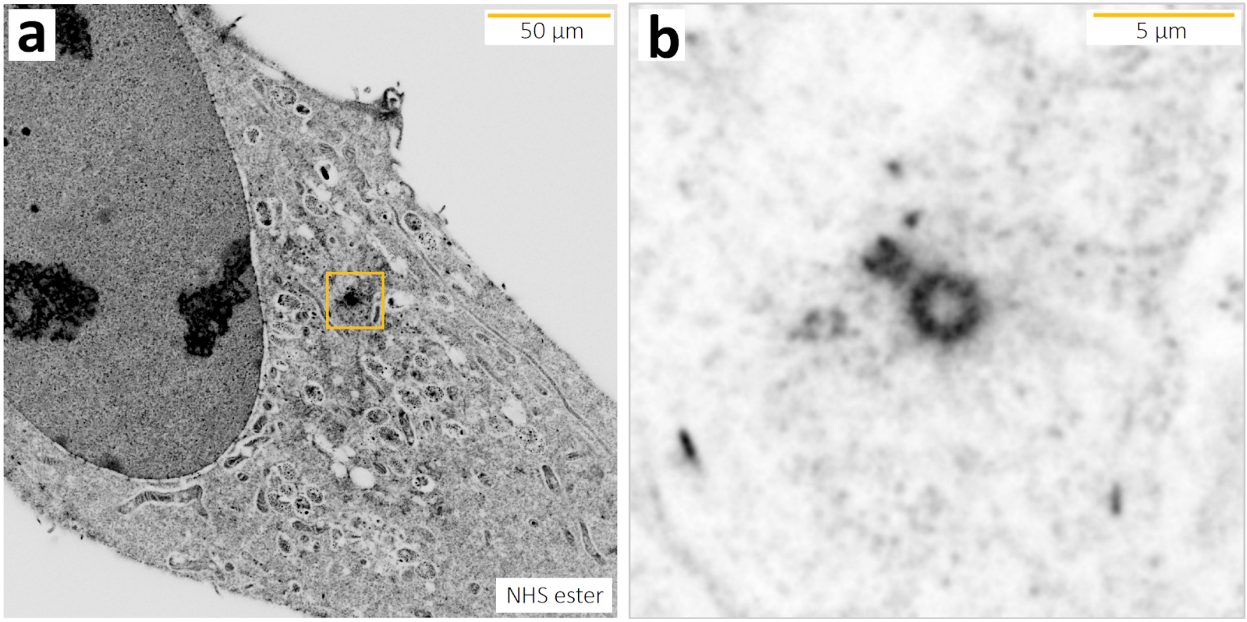

Supplementary Figure 13. pan-ExM reveals amine-dense centrosomes. a, *pan-ExM expanded HeLa cell pan-stained with NHS ester dye and imaged with a standard confocal microscope.* ***b***, *the magnified view of the area shown in the yellow box in a reveals the centrosome (adjusted for contrast). Representative images from 4 (a,b) independent experiments. The panels are displayed with a white-to-black color table. The yellow scale bars are not corrected for the expansion factor.*

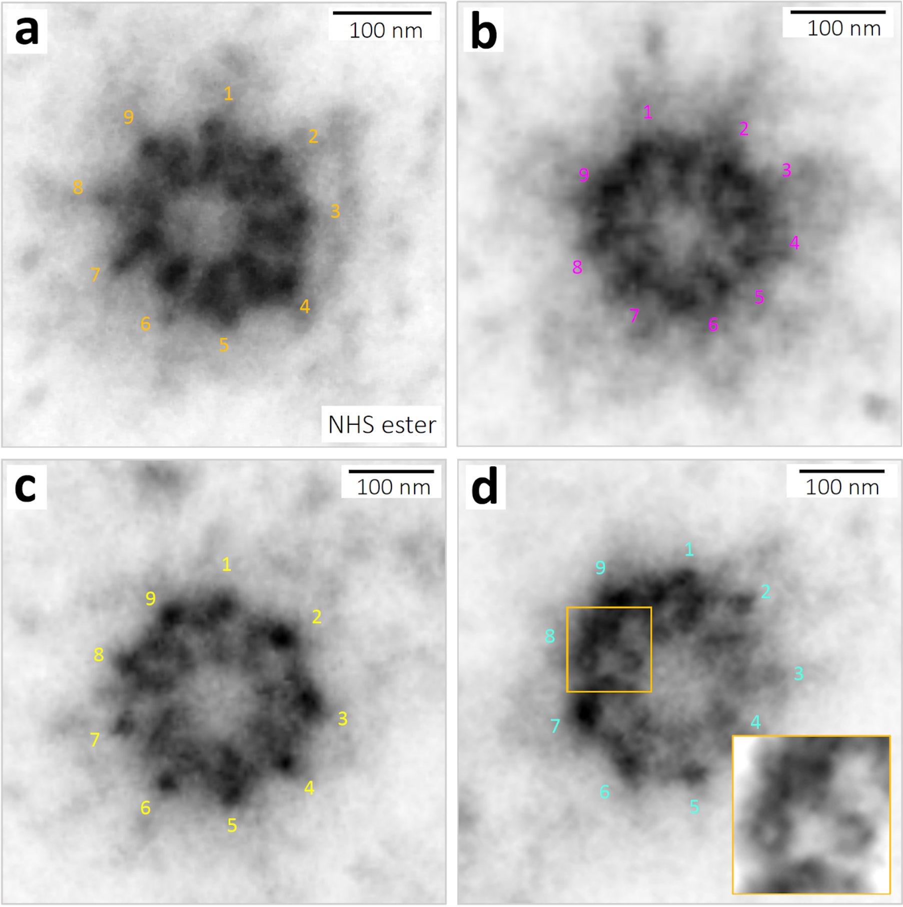

***Supplementary Figure 14. pan-ExM reveals centriole 9-fold symmetry. a-d**, Four centrioles processed with pan-ExM and pan-stained with NHS ester dye and imaged with a standard confocal microscope. Numbers denote microtubule triplets. The area in the yellow box in d is shown in inset and reveals rings resembling hollow microtubules within microtubule triplets. Representative images from 4 (a-d) independent experiments. Panels are displayed with a white-to-black color table. Scale bars are corrected for the expansion factor.*

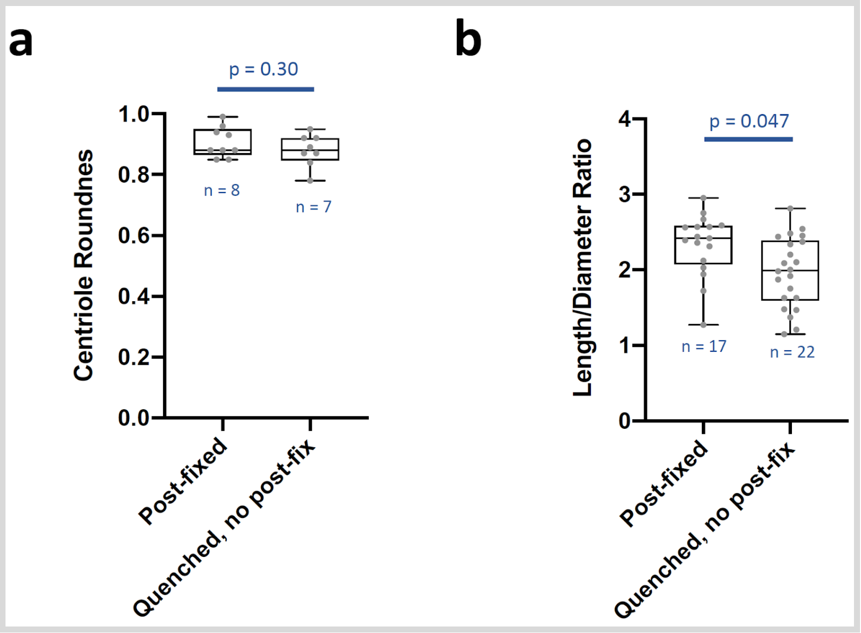

Supplementary Figure 15. Preventing sample crosslinking after the first expansion does not distort centriole roundedness and only minimally impacts the length-to-diameter ratio. a*, roundness measurements of centrioles in U-2OS cells processed with the standard pan-ExM protocol (Post-fixed: n = 8 centrioles) or with the pan-ExM modified protocol (see Methods; Quenched, no post-fix: n = 7 centrioles).* ***b****, length-to-diameter-ratio measurements of mature centrioles in U-2OS cells processed with the standard pan-ExM protocol (Post-fixed: n = 17 centrioles) or with the pan-ExM modified protocol (see Methods; Quenched, no post-fix: n = 22 centrioles). Medians and interquartile ranges are shown with whiskers drawn down to the minimum and maximum values. Means ± standard deviations are reported. An unpaired two-tailed t-test was used to analyze the data.*

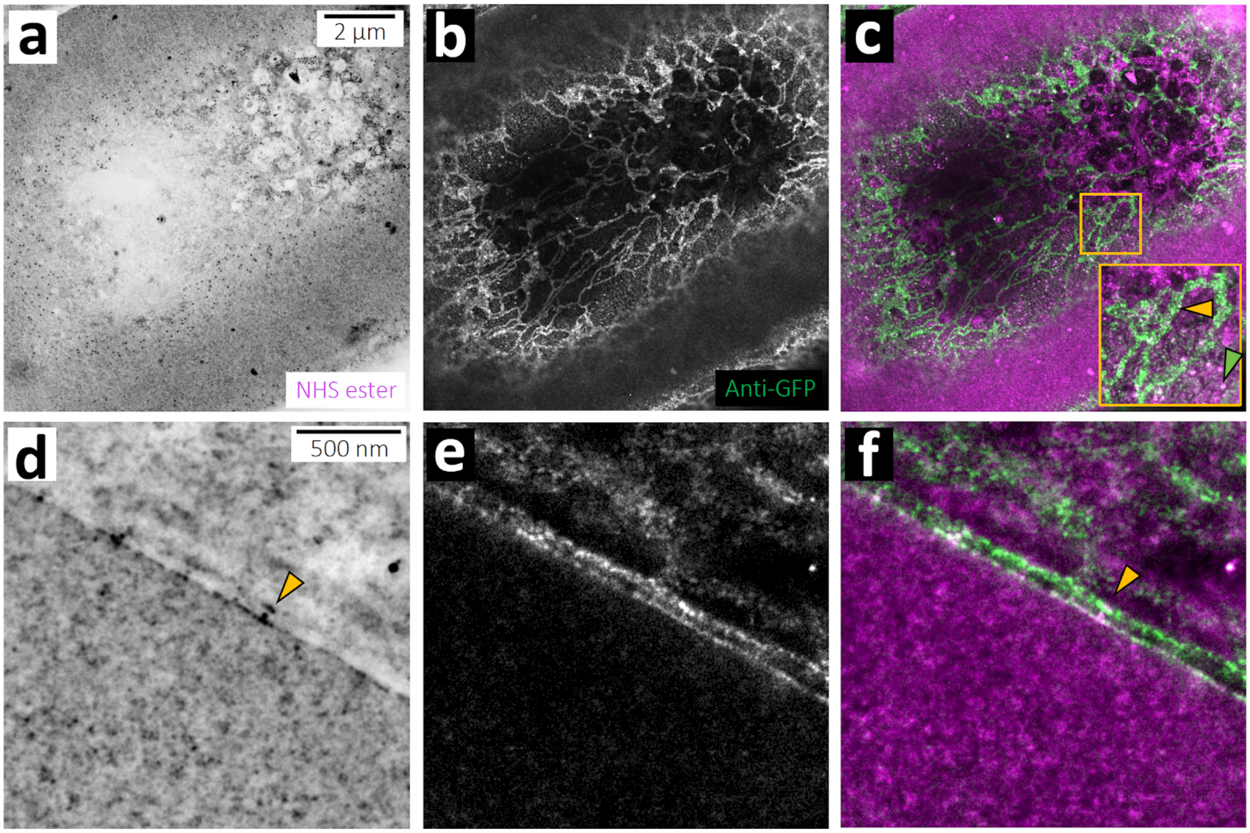

***Supplementary Figure 16. Imaging the ER in the context of cellular ultrastructure. a**, NHS ester pan-stained image of a HeLa cell expressing ER-membrane localized Sec61β-GFP, focusing on the bottom of the nucleus. **b**, same area as in a, showing anti-GFP immunostaining. **c**, overlay of a and b. The area in the yellow box is shown in the inset and reveals ER tubules (yellow arrowhead) and nuclear pore complexes (NPCs) (green arrowhead). **d**, NHS ester pan-stained image of a HeLa cell expressing ER-membrane localized Sec61β-GFP, showing the edge of the nucleus. The yellow arrowhead points at a NPC. **e**, same area as in d, showing anti-GFP immunostaining which reveals the two membranes of the nuclear envelope. **f**, overlay of d and e. The yellow arrowhead points at the same NPC as highlighted in d, showing that the staining is concentrated between the outer and inner membrane of the nuclear envelope. Representative images from 2 (a-f) independent experiments. Panels a and d are displayed with a white-to-black color table. Panels b and e are displayed with a black-to-white color table. Scale bars are corrected for the determined expansion factor. The shown images were recorded with a standard confocal microscope.*

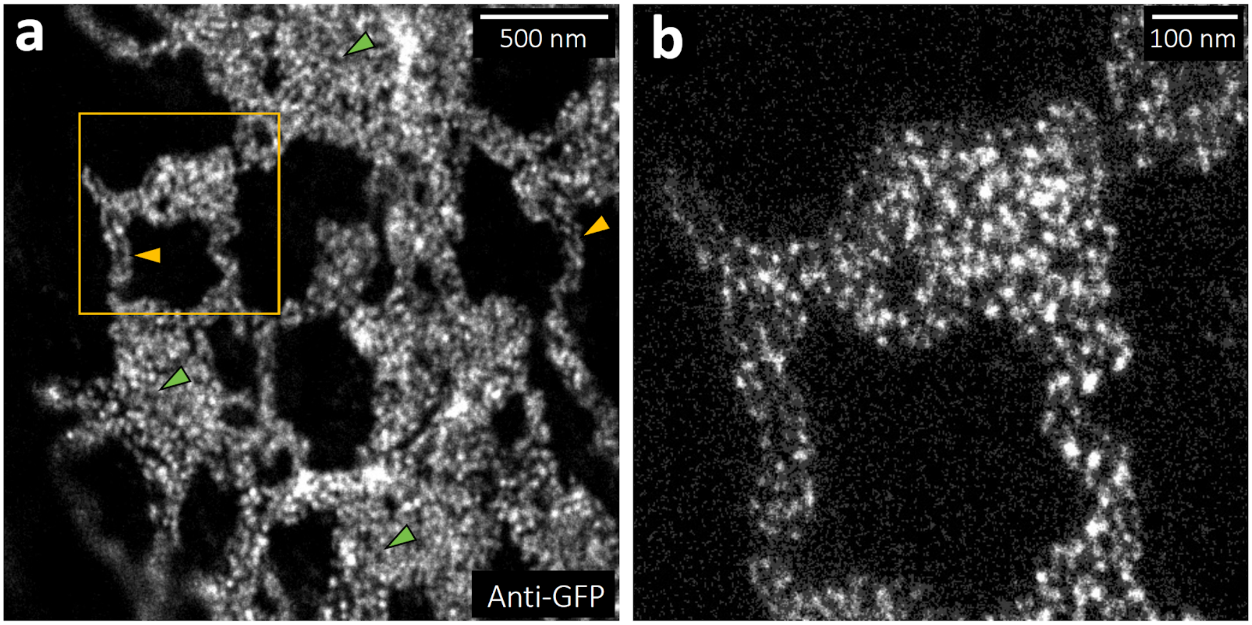

***Supplementary Figure 17. pan-ExM reveals ER architecture. a**, Anti-GFP signal in a HeLa cell expressing ER-membrane localized Sec61β-GFP, showing hollow ER tubules (yellow arrowheads) and fenestrated ER sheets (green arrowheads). The image was recorded in standard confocal mode. b, STED-super-resolution image of the area outlined by the yellow box in a showing clusters of Anti-GFP antibodies. Representative images from 2 (a) and 1 (b) independent experiments. The figures are displayed with a black-to-white color table. Scale bars are corrected for the determined expansion factor.*

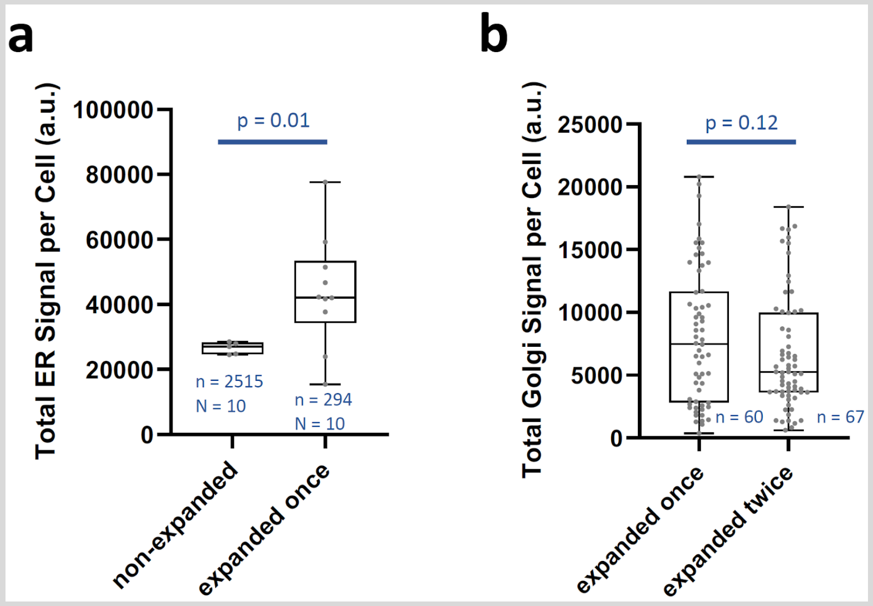

Supplementary Figure 18. Measurement of protein retention. ***a****, comparison of a non-expanded sample with a sample expanded once. (Non-expanded: n = 2515 cells, N = 10 FOVs; Expanded once: n = 294 cells, N = 10 FOVs.)* ***b****, comparison of a sample expanded once with a sample expanded twice. (Expanded once: n = 60 cells; Expanded twice: n = 67 cells). For each distribution, median and interquartile range are shown with whiskers drawn down to the minimum and maximum values. Means ± standard deviations are reported. An unpaired two-tailed t-test was used to analyze the data.*

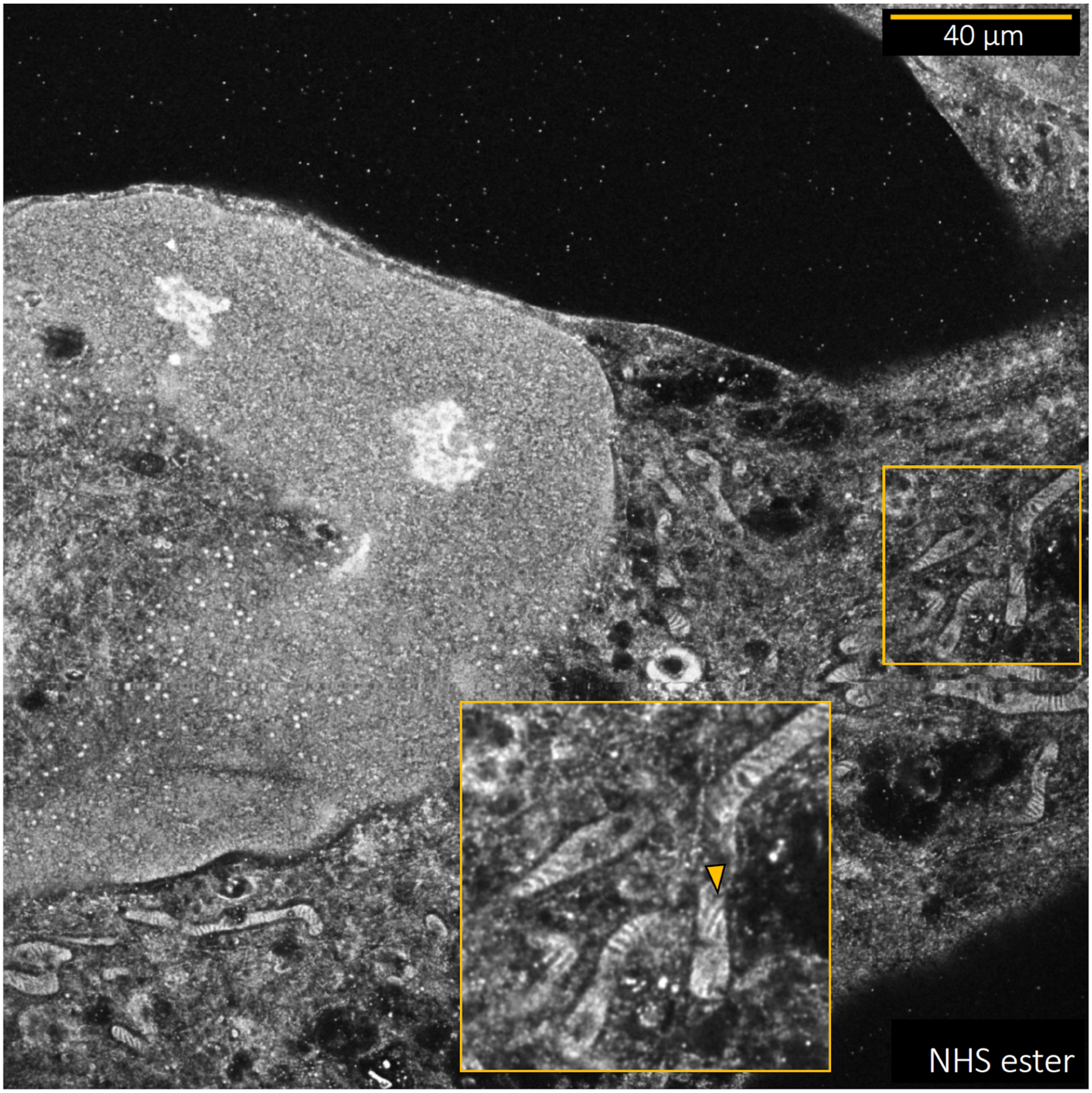

Supplementary Figure 19. HeLa cell processed with pan-ExM modified protocol (see Methods)*. The area in the yellow box is shown in the inset and reveals mitochondria cristae. Representative image from 1 independent experiment. The figure is displayed with black-to-white color table. The yellow scale bar is not corrected for the expansion factor. The shown image was recorded with a standard confocal microscope.*

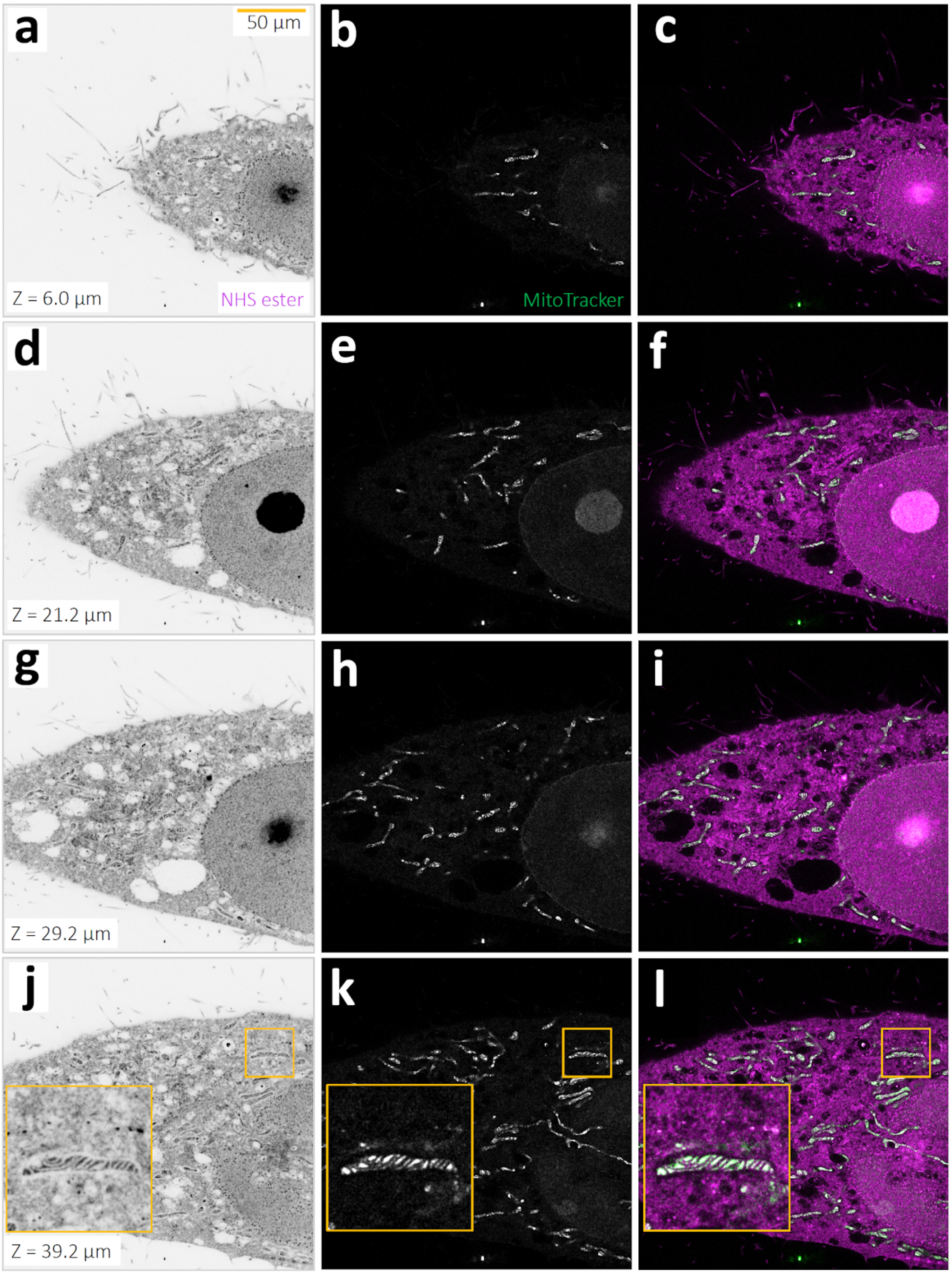

Supplementary Figure 20. 3D image stack of a HeLa cell pan-stained with NHS ester and labeled with MitoTracker Orange and imaged with a standard confocal microscope. ***a,d,g,j***, *NHS ester pan-stained images at axial positions 6.0, 21.2, 29.2 and 39.2 µm, respectively.* ***b,e,h,k***, *MitoTracker Orange staining of the same fields of view.* ***c,f,i,l***, *Overlay of the respective NHS ester and MitoTracker Orange images. The area outlined by the yellow boxes in j, k and l is shown in the insets and reveals mitochondrial cristae in both the NHS ester and MitoTracker Orange channels. Representative images from 5 (a-l) independent experiments. Panels a,b,d,e,g,h,j,k are displayed with a white-to-black color table. The scale bars and shown axial positions are not corrected for the determined expansion factor.*

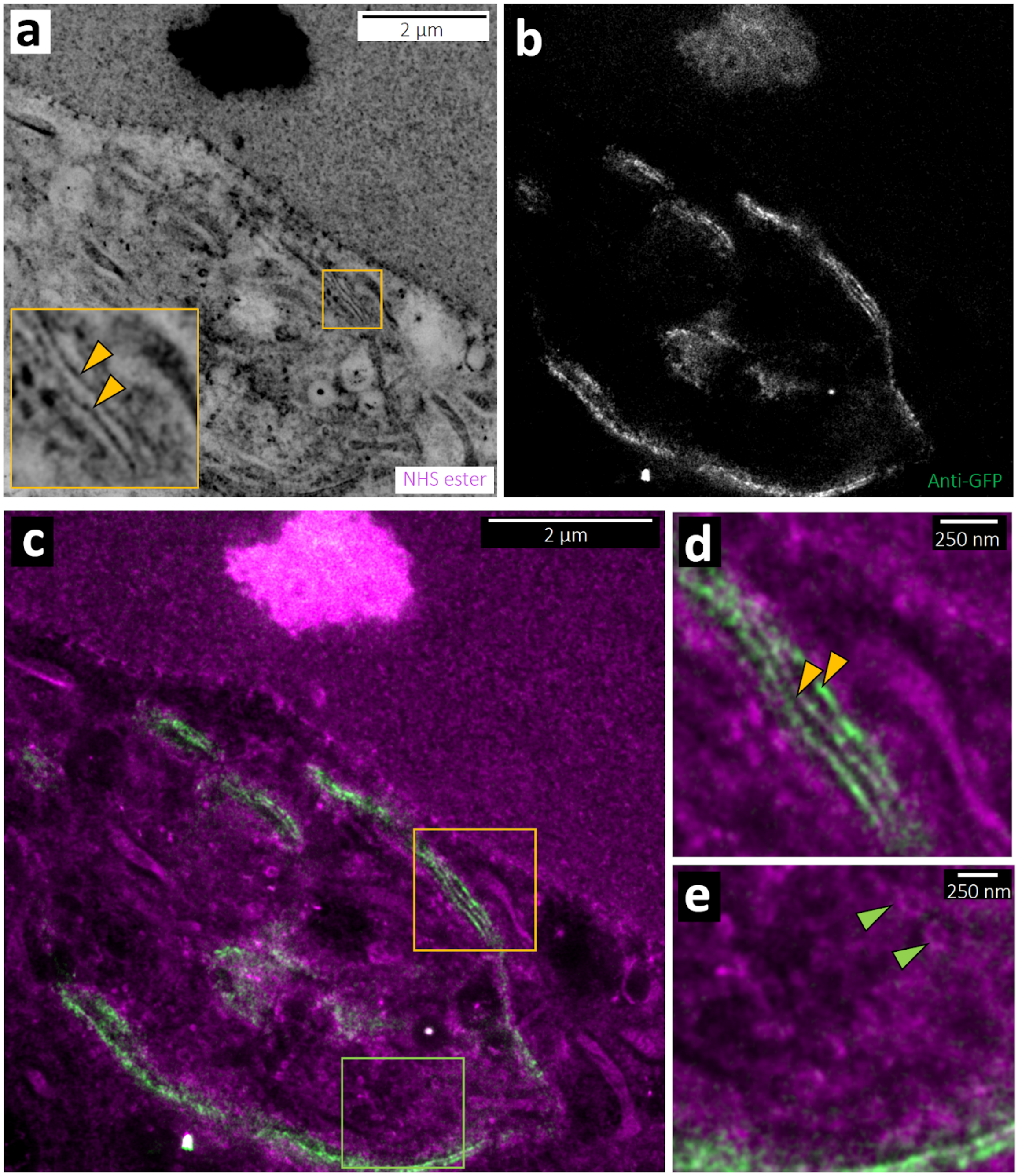

***Supplementary Figure 21: pan-ExM reveals perinuclear Golgi cisternae. a**, NHS ester pan-stained HeLa cell expressing Golgi-localized ManII-GFP. **b**, same area as in a, showing anti-GFP immunostaining. **c**, overlay of a and b. **d**, the area shown in the yellow box in c showing ManII-positive Golgi cisternae (yellow arrowheads). **e**, the area shown in the green box in c showing vesicle-like structures in the vicinity of the Golgi complex (green arrowheads). Representative images from 5 (a-e) independent experiments. Panel a is displayed with a white-to-black color table. Panel b is displayed with a black-to-white color table. Scale bars are corrected for the expansion factor. The shown images were recorded with a standard confocal microscope.*

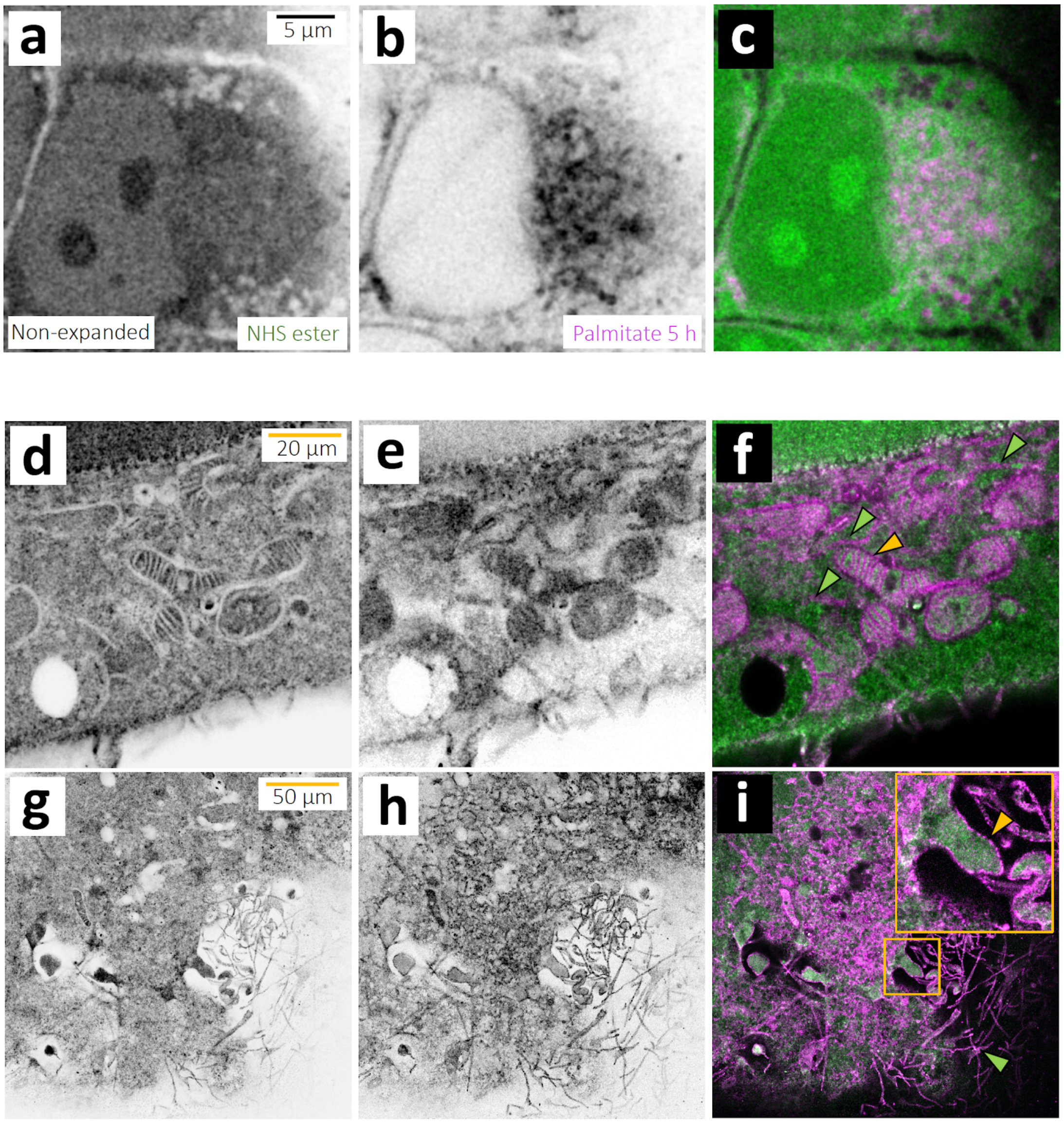

Supplementary Figure 22. Differential pan-staining reveals compartmentalized palmitate distribution in HeLa cells. a*, NHS ester pan-stained non-expanded HeLa cell.* ***b****, palmitate pan-staining of the same area.* ***c****, overlay of a and b.* ***d****, NHS ester pan-stained image of a pan-ExM expanded HeLa cell, showing the perinuclear cytoplasm.* ***e****, palmitate pan-staining of the same area.* ***f****, overlay of d and f. The yellow arrowhead points at palmitate-rich mitochondria cristae. Green arrowheads point at palmitoylated ER tubules.* ***g****, NHS ester pan-stained image of a pan-ExM expanded HeLa cell, showing the periphery of the cell.* ***h****, palmitate pan-staining of the same area.* ***i****, overlay of g and h. Representative images from 2 (a-i) independent experiments. The area outlined by the yellow box is shown in the inset and reveals a cross-section of the plasma membrane (yellow arrowhead). The green arrowhead points at palmitate-rich filopodia. Panels a,b,d,e,g,h are displayed with a white-to-black color table. Yellow scale bars are not corrected for the expansion factor. The shown images were recorded with a standard confocal microscope.*

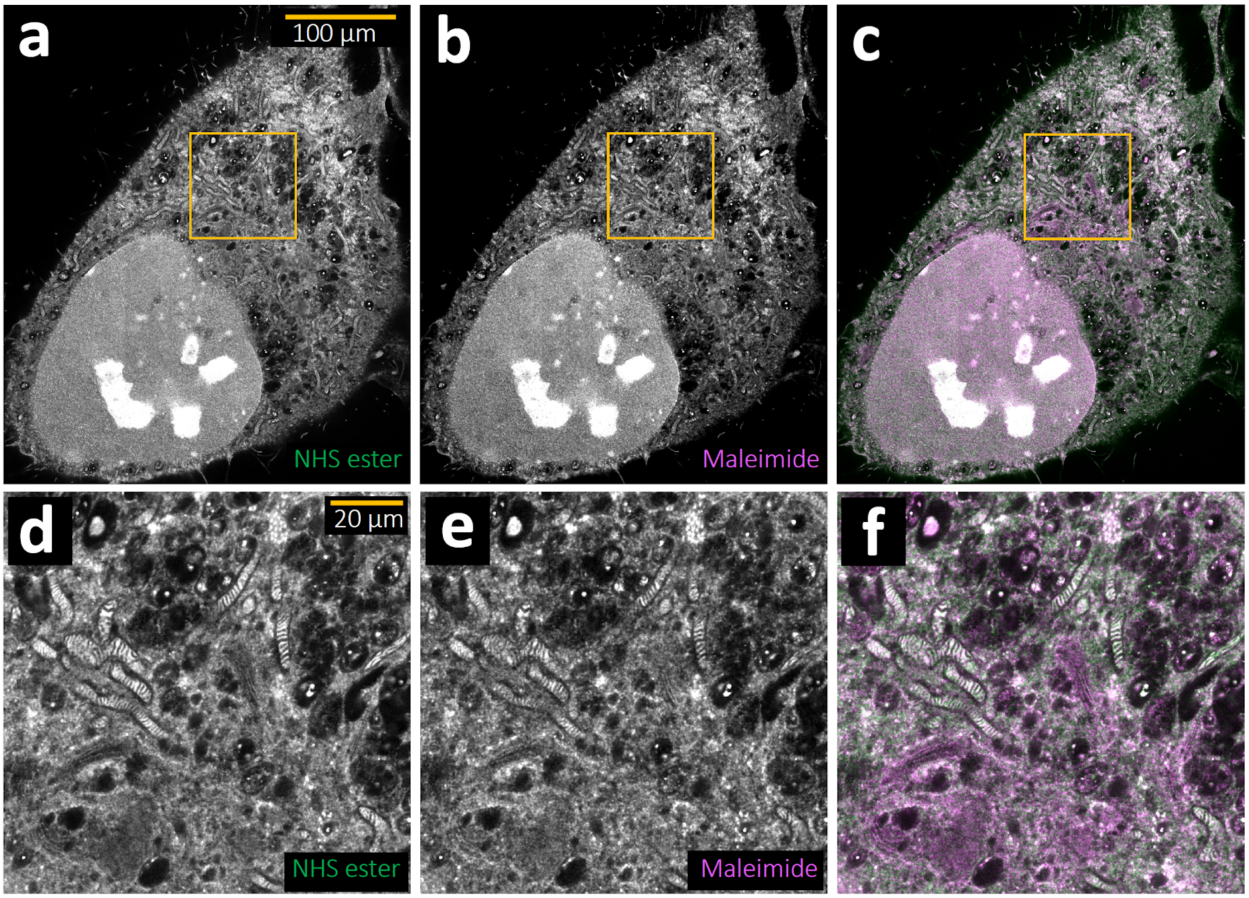

***Supplementary Figure 23. pan-ExM reveals cellular ultrastructure in a HeLa cell. a**, NHS ester pan-staining channel. **b**, Maleimide pan-staining channel. **c**, overlay of a and b. **d-f**, the area in the yellow boxes shown in a-c, respectively. Representative images from 2 (a-f) independent experiments. All panels are displayed with a black-to-white color table. Yellow scale bars are not corrected for the expansion factor. The shown images were recorded with a standard confocal microscope.*

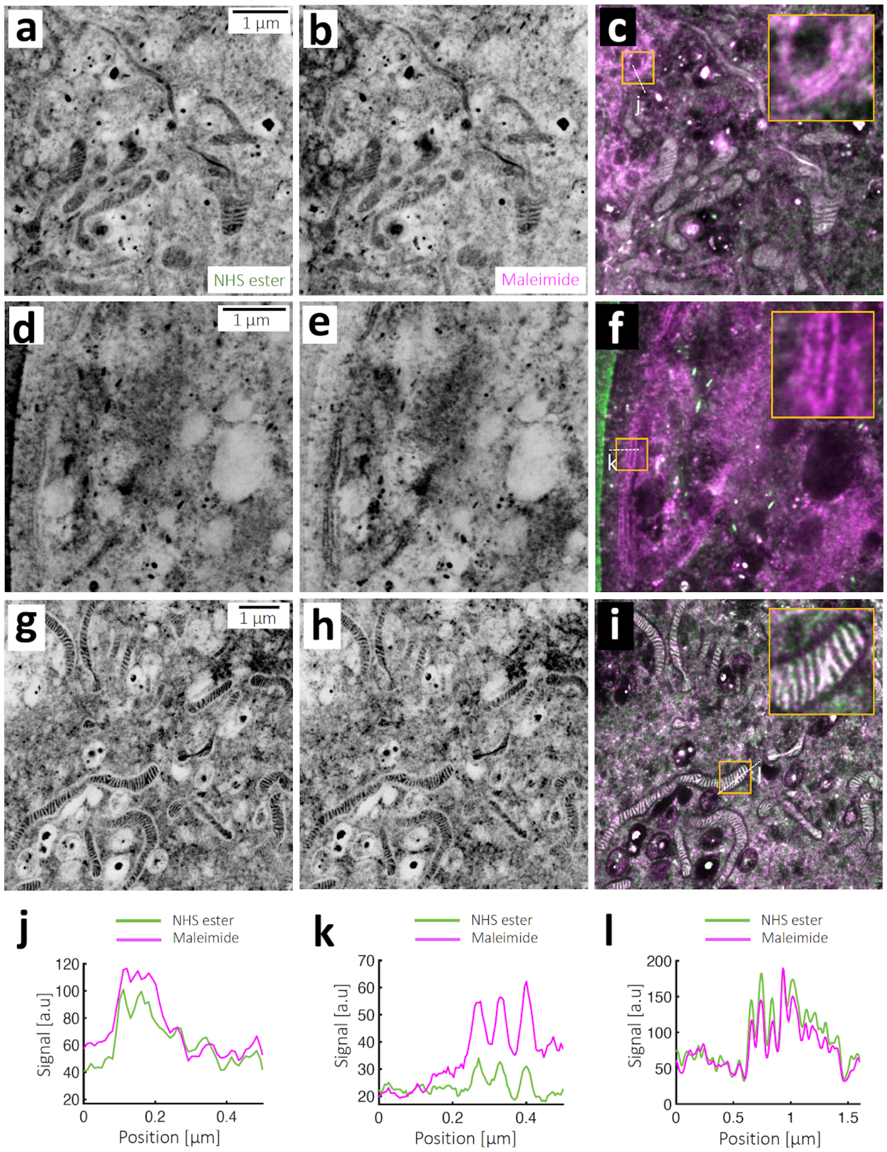

***Supplementary Figure 24. Differential pan-staining reveals the distribution of the cysteine proteome in HeLa cells. a**, NHS ester pan-stained HeLa cell. **b**, maleimide channel of the same area. **c**, overlay of a and b. The area in the yellow box in c shows cysteine-enriched Golgi cisternae. **d**, NHS ester pan-stained HeLa cell. **e**, maleimide channel of the same area. **f**, overlay of d and e. The area in the yellow box in f shows cysteine-enriched Golgi cisternae near the nucleus. **g**, NHS ester pan-stained HeLa cell. **h**, Maleimide channel of the same area. **f**, overlay of g and h. The area in the yellow box in i shows mitochondria with a nearly constant lysine to cysteine labeling ratio. Representative images from 2 (a-i) independent experiments. **j,k**, line profiles along the dashed lines in c and f respectively, revealing the change in NHS ester to maleimide staining across the Golgi. **l**, line profile along the dashed lines in i, revealing the change in NHS ester to maleimide staining across the cytosol and a mitochondrion. Panels a,b,d,e,g,h are displayed with a white-to-black color table. Scale bars are corrected for the expansion factor. The shown images were recorded with a standard confocal microscope.*

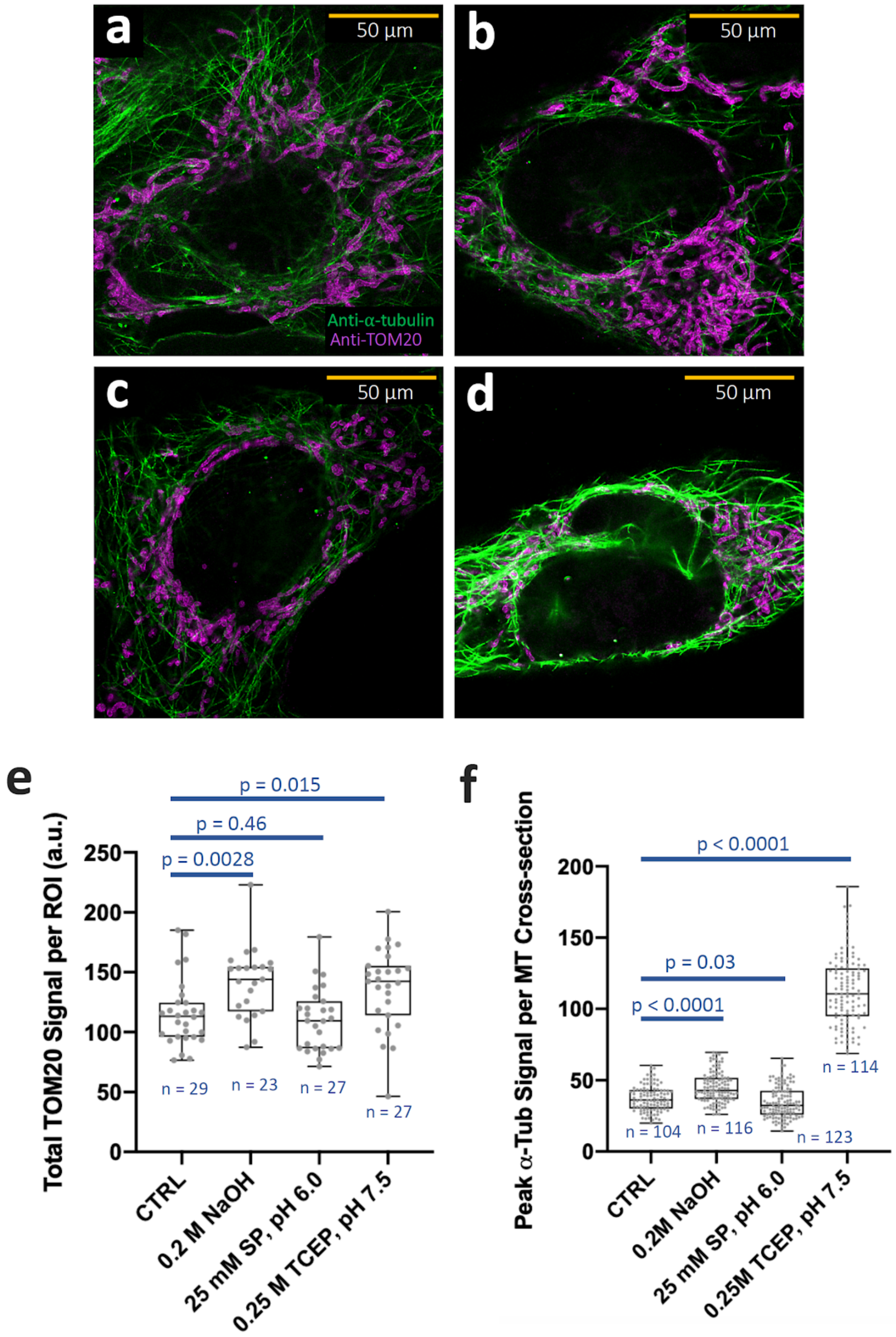

Supplementary Figure 25. Quantification of antibody labeling efficiency. *Anti-TOM20 and anti-α-tubulin staining in once-expanded N,N'-methylenebisacrylamide crosslinked gels: without further treatment after denaturation (CTRL; image shown in* ***a****); treated with 0.2 M NaOH for 1 hour after denaturation (0.2 M NaOH; image shown in* ***b****); treated with 25 mM sodium periodate (SP) in 100 mM acetate buffer, pH 6.0 for 1 hour after denaturation (25 mM SP, pH 6.0; image shown in* ***c****); treated with 0.25 M TCEP in 1 M Tris-Cl buffer, pH 7.5 for 18 hours after denaturation (0.25 M TCEP, pH 7.5; image shown in* ***d****). Representative images from 5 cells from 1 independent experiment (a-d).* ***e****, comparison of anti-TOM20 signal for these four conditions (CTRL: n = 29 FOVs, N = 5 cells; 0.2 M NaOH: n = 23 FOVs, N = 5 cells; 25 mM SP,*

pH 6.0: n = 27 FOVs, N = 5 cells; 0.25 M TCEP, pH 7.5: n = 27 FOVs, N = 5 cells). ***f**, comparison anti-α-tubulin signal for the same four conditions (CTRL: n = 104 measurements, N = 5 cells; 0.2 M NaOH: n = 116 measurements, N = 5 cells; 25 mM SP, pH 6.0: n = 123 measurements, N = 5 cells; 0.25 M TCEP, pH 7.5: n = 114 measurements, N = 5 cells). Median and interquartile range are shown with whiskers drawn down to the minimum and maximum values. Means ± standard deviations are reported. An unpaired two-tailed t-test was used to compare the mean value of CTRL to the mean of each of the three treatments in e,f. Yellow scale bars are not corrected for the expansion factor.*

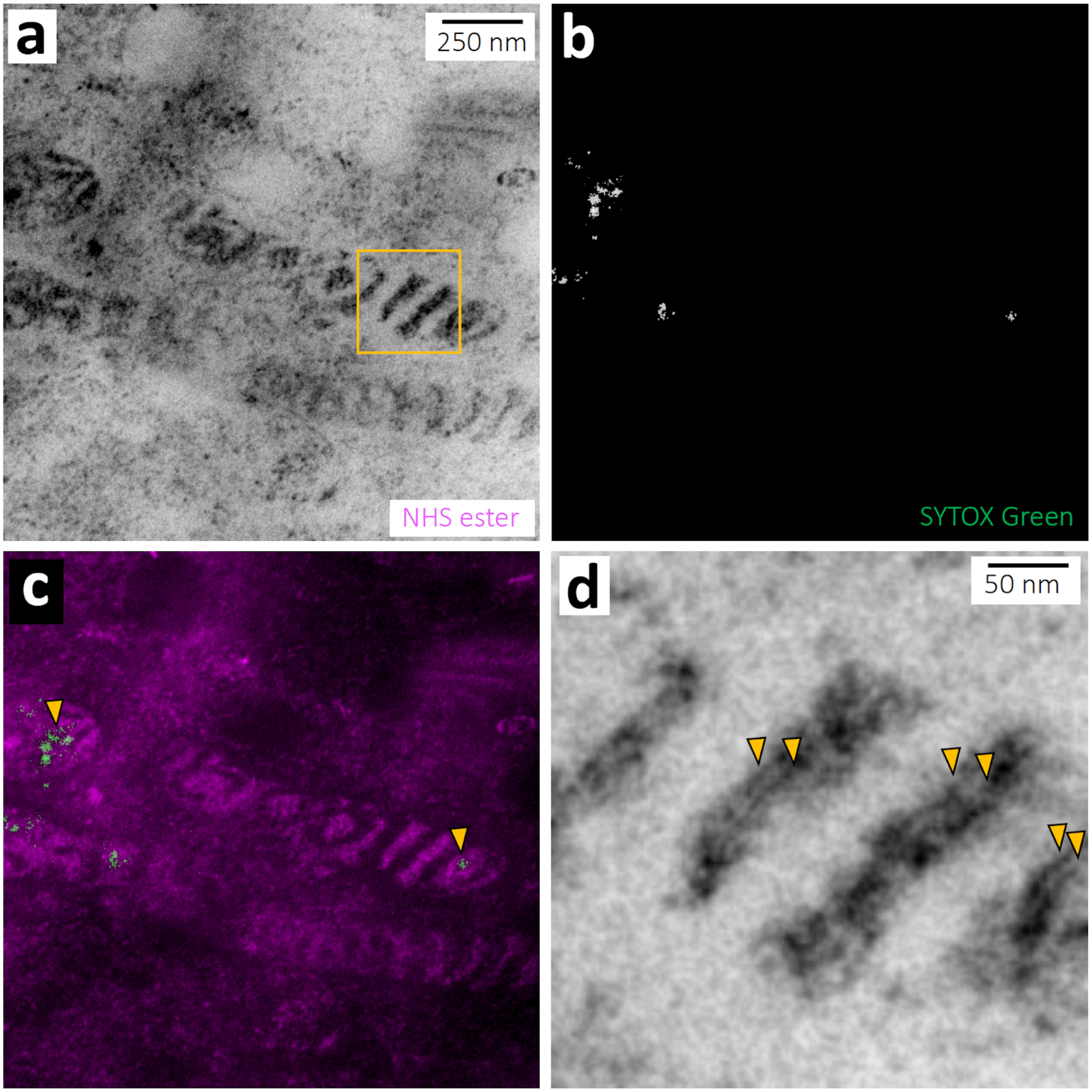

Supplementary Figure 26. pan-ExM combined with STED microscopy reveals mitochondria ultrastructure. a, *NHS ester pan-stained HeLa cell showing mitochondria and imaged with STED super-resolution microscopy.* ***b***, *SYTOX Green staining in same area.* ***c***, *overlay of a and b. Yellow arrowheads point at mitochondrial DNA (mtDNA) nucleoids.* ***d***, *magnified view of the area in the yellow box in a showing amine-rich staining on two sides of mitochondria cristae. Representative images from 2 (a-d) independent experiments. Panels a and d are displayed with a white-to-black color table. Panel b is displayed with a black-to-white color table. Scale bars are corrected for the expansion factor.*

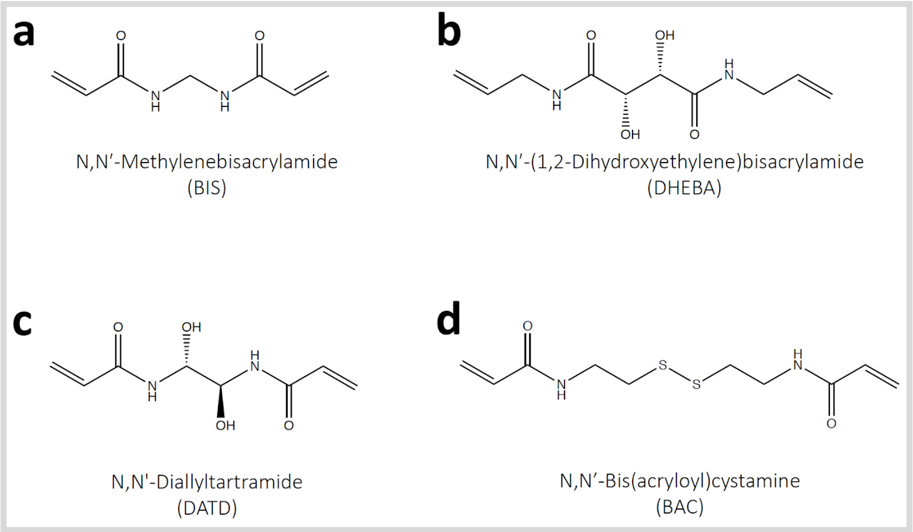

***Supplementary Figure 27. Chemical structures of several commercially available crosslinkers. a**, N,N'-Methylenebisacrylamide (BIS), a standard acrylamide crosslinker. **b**, N,N'-(1,2-Dihydroxyethylene)bisacrylamide (DHEBA), a crosslinker whose amidomethylol bonds are readily cleaved with base hydrolysis. **c**, N,N'-Diallyltartramide (DATD), a crosslinker whose diol bond Is readily cleaved with periodate. **d**, N,N'-Bis(acryloyl)cystamine (BAC), a crosslinker whose disulfide bond is cleaved with reducing agents.*

Step	Reagent	Acronym	Storage	Vendor	Catalog number
Gelations	40% Acrylamide	AAm	4°C	Sigma-Aldrich	A9099
	N,N'-(1,2-Dihydroxyethylene)bisacrylamide	DHEBA	RT		294381
	Sodium hydroxide	NaOH	RT		S8045
	Sodium acrylate	SA	-20°C in desiccator	Santa Cruz Biotechnology	sc-236893C
	N,N'-Methylenebis(acrylamide)	BIS	RT	Alfa Aesar	J66710
	Ammonium persulfate	APS	RT in desiccator	American Bio	AB00112
	N,N,N',N'-Tetramethylethylenediamine	TEMED	RT in desiccator		AB02020
	Tris [hyroxymethyl] aminomethane	Tris	RT		AB02000
	20% Sodium dodecyl sulfate solution	SDS	RT		AB01922
	Sodium chloride	NaCl	RT	J.T. Baker	3624-01
	1x Phosphate buffered saline (Gibco)	1X PBS	RT	Thermofisher	10010023
	10x Phosphate buffered saline (Gibco)	10X PBS	RT		70011044
Cell culture & fixation	Potassium hydroxide	KOH	RT	Macron Fine Chemicals	6984-04
	Fibronectin solution	-	4°C	Sigma-Aldrich	F1141
	16% Paraformaldehyde	FA	RT	Electron Microscopy Sciences	15710
	8% Glutaraldehyde	GA	4°C		16019
**Pan-staining and labeling**	Ca^{++} and Mg^{++} free HBSS buffer (Gibco)	HBSS	RT	Thermofisher	14170112
	SYTOX Green	-	-20°C		S7020
	Click-IT™ Palmitic Acid, Azide	-	-20°C		C10265
	MitoTracker Orange CMTMRos (Invitrogen)	-	-20°C		M7510
	Bovine Serum Albumin	BSA	4°C	Jackson ImmunoResearch	001-000-162
	Sodium bicarbonate	$NaHCO_3$	RT	Sigma-Aldrich	S5761
	Tween 20	-	RT		P7949
	NHS ester-ATTO594	-	-20°C		08741
	NHS ester-ATTO532	-	-20°C		88793
	Maleimide-ATTO594	-	-20°C		08717
	Tris(2-carboxyethyl)phosphine hydrochloride	TCEP	RT		646547
	Alkyne-ATTO590	-	-20°C		93990
	Fatty acid free Bovine Serum Albumin	Delipidated BSA	4°C		A4612
	NHS ester-DY634	-	-20°C	Dyomics	634-01A
Antibodies	Rabbit polyclonal anti-TOM20	Anti-TOM20	-20°C	Abcam	ab78547
	Rabbit polyclonal anti-GFP (Invitrogen)	Anti-GFP	4°C	Thermofisher	A11122
	Rabbit polyclonal anti-polyglutamate chain	Anti-PolyE	-20°C	Adipogen	AG-25B-0030-C050
	Monoclonal mouse anti-α-tubulin	-	-20°C	Sigma-Aldrich	T6199 or T5168
	ATTO647N-conjugated anti-mouse	-	-20°C		50185
	ATTO647N-conjugated anti-rabbit	-	-20°C		40839
	ATTO594-conjugated anti-rabbit	-	-20°C		77671

Supplementary Table 1. Used reagents

Materials	Vendor	Catalog number
No. 1.5 12-mm round glass coverslips	Electron Microscopy Sciences	72230-01
Glass microscope slide	Sigma-Aldrich	S8400
No. 1.5 22 x 22 mm square cover glass coverslips	Fisher Scientific	12-541BP
No. 1.5 18-mm round coverslip	Marienfeld	0117580
50 mm MatTek dish, No. 1.5 coverslip 30-mm diameter	MatTek Life Sciences	P50G-1.5-30-F
Picodent Twinsil	Picodent	1300 1000

Supplementary Table 2. Used materials

Instruments	Sonic bath, pH meter, 37°C incubator, nitrogen (or argon) gas tank, dry block incubator, ice bucket, rocker, vortex
Other materials	Superglue, tweezers, razor blades, container (plastic Tupperware), 24-well plates, 6-well plates, 1.5 mL Eppendorf tubes, 15 mL Eppendorf tubes, 50 mL Eppendorf tubs, Petri dishes, 30-mm dishes, Kim wipes, beaker, stir bar, paintbrush (brush size No. 2 (1.6 mm)), laminated tape, pH strips, 100% ethanol, aluminum foil, surgical scissors

Supplementary Table 3. Used instruments, tools and disposables
