# Supplementary material for: Light microscopy of proteins in their ultrastructural context
Source: Nat Commun. 2020 Jul 31;11:3850. doi: 10.1038/s41467-020-17523-8 (PMC7395138; doi:10.1038/s41467-020-17523-8)
Supplement: Supplementary file 2 — Description of Additional Supplementary Files [file 41467_2020_17523_MOESM2_ESM.pdf]

Description of Additional Supplementary Files

File Name: Supplementary Movie 1
Description: Animation of 3D data set showing the centriole of Figure 5a-c. The data set was recorded with a 63x/1.2 NA water immersion objective on a conventional confocal microscope (Leica TCS SP8).

File Name: Supplementary Movie 2
Description: Animation of 3D data set showing a centriole in axial orientation. The data set was recorded with a 63x/1.2 NA water immersion objective on a conventional confocal microscope (Leica TCS SP8).

File Name: Supplementary Movie 3
Description: Animation of a large (153 slices) 3D stack showing a HeLa cell with NHS ester pan-staining recorded with a 63x/1.2 NA water immersion objective on a conventional confocal microscope (Leica TCS SP8).

File Name: Supplementary Movie 4
Description: Animation of a large (103 slices) 3D stack showing a HeLa cell with NHS ester pan-staining and Mitotracker Orange. The data set was recorded with a 63x/1.2 NA water immersion objective on a conventional confocal microscope (Leica TCS SP8). The video shows first the NHS ester pan-staining channel, then the Mitotracker Orange channel and in the end the overlay of the two with NHS ester pan-staining displayed in magenta and Mitotracker Orange in green.

File Name: Supplementary Movie 5
Description: Animation of the 3D data set shown in Fig. 7. The data set was recorded with a 63x/1.4 NA oil immersion objective on a conventional confocal microscope (Leica TCS SP8)
