# Supplementary material for: Light microscopy of proteins in their ultrastructural context
Source: Nat Commun. 2020 Jul 31;11:3850. doi: 10.1038/s41467-020-17523-8 (PMC7395138; doi:10.1038/s41467-020-17523-8)
Supplement: Supplementary file 8 — Reporting Summary [file 41467_2020_17523_MOESM8_ESM.pdf]

nature research

Corresponding author(s): Joerg Bewersdorf

Last updated by author(s): Jun 27, 2020

Reporting Summary

Nature Research wishes to improve the reproducibility of the work that we publish. This form provides structure for consistency and transparency in reporting. For further information on Nature Research policies, see Authors & Referees and the Editorial Policy Checklist .

Statistics

For all statistical analyses, confirm that the following items are present in the figure legend, table legend, main text, or Methods section.

n/a	Confirmed	
☐	☒	The exact sample size (*n*) for each experimental group/condition, given as a discrete number and unit of measurement
☐	☒	A statement on whether measurements were taken from distinct samples or whether the same sample was measured repeatedly
☐	☒	The statistical test(s) used AND whether they are one- or two-sided<br>*Only common tests should be described solely by name; describe more complex techniques in the Methods section.*
☒	☐	A description of all covariates tested
☒	☐	A description of any assumptions or corrections, such as tests of normality and adjustment for multiple comparisons
☐	☒	A full description of the statistical parameters including central tendency (e.g. means) or other basic estimates (e.g. regression coefficient) AND variation (e.g. standard deviation) or associated estimates of uncertainty (e.g. confidence intervals)
☐	☒	For null hypothesis testing, the test statistic (e.g. *F*, *t*, *r*) with confidence intervals, effect sizes, degrees of freedom and *P* value noted<br>*Give P values as exact values whenever suitable.*
☒	☐	For Bayesian analysis, information on the choice of priors and Markov chain Monte Carlo settings
☒	☐	For hierarchical and complex designs, identification of the appropriate level for tests and full reporting of outcomes
☒	☐	Estimates of effect sizes (e.g. Cohen's *d*, Pearson's *r*), indicating how they were calculated

Our web collection on statistics for biologists contains articles on many of the points above.

Software and code

Policy information about availability of computer code

Data collection	No custom computer code was used.
Data analysis	Confocal microscopy data was recorded using Leica Application Suite X (version 3.5.2.18963) software. Data analysis was performed using ImageJ/FIJI (version 2.0.0-rc-69) and the commercially available Imspector program (version 1,0,0,4). RMS error calculations and plotting were performed using a B-spline-based non-rigid registration (version 1.33.0.0) MatLab (version R2020a) package. Graphpad Prism version 8 was used for statistical analyses.

For manuscripts utilizing custom algorithms or software that are central to the research but not yet described in published literature, software must be made available to editors/reviewers. We strongly encourage code deposition in a community repository (e.g. GitHub). See the Nature Research guidelines for submitting code & software for further information.

Data

Policy information about availability of data

All manuscripts must include a data availability statement. This statement should provide the following information, where applicable:
- Accession codes, unique identifiers, or web links for publicly available datasets
- A list of figures that have associated raw data
- A description of any restrictions on data availability

The datasets generated and/or analyzed during the current study are available from the corresponding author on request.

Field-specific reporting

Please select the one below that is the best fit for your research. If you are not sure, read the appropriate sections before making your selection.

Life sciences study design

All studies must disclose on these points even when the disclosure is negative.

Sample size	This is a methods paper describing a method development. We imaged at least 5 cells per experiment. Our sample size was selected to adequately demonstrate the technical performance of our method and was limited by an acceptable microscope usage time.
Data exclusions	Samples showing inadequate fluorescent labeling quality or macroscale distortions were excluded from the analysis. These exclusion criteria were pre-established.
Replication	The following images are representatives of at least three independent experiments: Fig. 1-7 (except 5b, 6b-e), Suppl. Fig. 5-9, 11b, 13, 14, 20, 21. The following images are representatives of two independent experiments: Fig. 8, Suppl. Fig. 22, 23, 24, 26. Fig. 6b-e, Suppl. Fig. 16, 17 were replicated once using slightly different gelation chemistry. Suppl. Fig. 10 reports an unexpected observation and no replication has been attempted since it is very rare to observe a cell in cytokinesis in expanded gels. The experiments represented in Fig. 5c and Suppl. Fig. 19 have been performed once. Replication of these experiments has not been attempted because of the COVID-19 related lock-down of the laboratory. In some cases (Suppl. Fig. 11a, Suppl. Fig. 12, 15, 18, 25), multiple cells or fields of view (as stated in the figure captions) coming from the same gel were measured. All performed attempts of replication were successful.
Randomization	No randomization was performed. Randomization was considered to not be necessary as the purpose of the study was to demonstrate a new technique and not report biological results.
Blinding	No blinding was performed. Blinding was considered to not be necessary as the purpose of the study was to demonstrate a new technique and not report biological results.

Reporting for specific materials, systems and methods

We require information from authors about some types of materials, experimental systems and methods used in many studies. Here, indicate whether each material, system or method listed is relevant to your study. If you are not sure if a list item applies to your research, read the appropriate section before selecting a response.

Materials & experimental systems

n/a	Involved in the study	
☐	☒	Antibodies
☐	☒	Eukaryotic cell lines
☒	☐	Palaeontology
☒	☐	Animals and other organisms
☒	☐	Human research participants
☒	☐	Clinical data

Methods

n/a	Involved in the study	
☒	☐	ChIP-seq
☒	☐	Flow cytometry
☒	☐	MRI-based neuroimaging

Antibodies

Antibodies used	Rabbit polyclonal anti-TOM20 (Abcam, cat. no. ab78547)<br>Rabbit polyclonal anti-GFP (Invitrogen, A11122)<br>Rabbit polyclonal anti-polyglutamate chain [IN105] (Adipogen, cat. no. AG-25B-0030-C050)<br>Monoclonal mouse anti-α-tubulin [DM1alpha] (Sigma, cat. no. T6199)<br>Monoclonal mouse anti-α-tubulin [B-5-1-2] (Sigma, cat. no. T5168)<br>ATTO647N-conjugated anti-mouse IgG (Sigma, cat. no. 50185)<br>ATTO647N-conjugated anti-rabbit IgG (Sigma, cat. no. 40839)<br>ATTO594-conjugated anti-rabbit IgG (Sigma, cat. no. 77671)
Validation	In addition to the validation information provided on the manufacturers' websites, we verified that antibodies exhibited the expected sub-cellular localization in immuno-fluorescence images. More explicitly:<br>Anti-TOM20 antibody recognizes a 'synthetic peptide corresponding to Human TOMM20 aa 100 to the C-terminus (C terminal) conjugated to keyhole limpet haemocyanin' (https://www.abcam.com/tomm20-antibody-mitochondrial-marker-ab78547.html)<br>Anti-PolyE [IN105] recognizes 'C-terminally located linear alpha-glutamate chains of 4 and more glutamate residue' (https://adipogen.com/ag-25b-0030-anti-polyglutamate-chain-polye-pab-in105.html/)<br>Anti-α-tubulin [DM1alpha] recognizes an epitope located at the C-terminal end of the α-tubulin isoform (amino acids 426-430) (https://www.sigmaaldrich.com/catalog/product/sigma/t6199?lang=en&region=US)<br>Anti-α-tubulin [B-5-1-2] recognizes 'an epitope located at the C-terminal end of the α-tubulin isoform' (https://www.sigmaaldrich.com/catalog/product/sigma/t6199?lang=en&region=US)<br>Anti-GFP recognizes GFP that was isolated directly from the jellyfish Aequorea victoria.(https://www.thermofisher.com/antibody/product/GFP-Antibody-Polyclonal/A-11122)

Eukaryotic cell lines

Policy information about cell lines

Cell line source(s)	HeLa (CCL-2; ATCC)<br>U2OS (HTB-96; ATCC)
Authentication	Cell lines were purchased from ATCC and showed the expected morphological features. No further authentication was performed.
Mycoplasma contamination	Cell lines were not tested for mycoplasma contamination.
Commonly misidentified lines<br>(See ICLAC register)	No ICLAC cell lines were used.
